# The value of confirmatory testing in early infant HIV diagnosis programmes in South Africa: A cost-effectiveness analysis

**DOI:** 10.1371/journal.pmed.1002446

**Published:** 2017-11-21

**Authors:** Lorna Dunning, Jordan A. Francke, Divya Mallampati, Rachel L. MacLean, Martina Penazzato, Taige Hou, Landon Myer, Elaine J. Abrams, Rochelle P. Walensky, Valériane Leroy, Kenneth A. Freedberg, Andrea Ciaranello

**Affiliations:** 1 Division of Epidemiology and Biostatistics, School of Public Health & Family Medicine, University of Cape Town, Cape Town, South Africa; 2 Medical Practice Evaluation Centre, Department of Medicine, Massachusetts General Hospital, Boston, Massachusetts, United States of America; 3 Department of Obstetrics and Gynecology, Northwestern University, Chicago, Illinois, United States of America; 4 Department of HIV/AIDS, World Health Organization, Geneva, Switzerland; 5 Centre for Infectious Diseases Epidemiology & Research, School of Public Health & Family Medicine, University of Cape Town, Cape Town, South Africa; 6 ICAP at Columbia University, Mailman School of Public Health, Columbia University, New York, New York, United States of America; 7 College of Physicians & Surgeons, Columbia University, New York, New York, United States of America; 8 Division of Infectious Diseases, Department of Medicine, Massachusetts General Hospital, Boston, Massachusetts, United States of America; 9 Division of Infectious Diseases, Brigham and Women’s Hospital, Boston, Massachusetts, United States of America; 10 Center for AIDS Research, Harvard University, Boston, Massachusetts, United States of America; 11 Inserm, U1027, University of Toulouse 3, Toulouse, France; 12 Department of Health Policy and Management, Harvard T.H. Chan School of Public Health, Boston, Massachusetts, United States of America; Elizabeth Glaser Pediatric AIDS Foundation, UNITED STATES

## Abstract

**Background:**

The specificity of nucleic acid amplification tests (NAATs) used for early infant diagnosis (EID) of HIV infection is <100%, leading some HIV-uninfected infants to be incorrectly identified as HIV-infected. The World Health Organization recommends that infants undergo a second NAAT to confirm any positive test result, but implementation is limited. Our objective was to determine the impact and cost-effectiveness of confirmatory HIV testing for EID programmes in South Africa.

**Method and findings:**

Using the Cost-effectiveness of Preventing AIDS Complications (CEPAC)–Pediatric model, we simulated EID testing at age 6 weeks for HIV-exposed infants without and with confirmatory testing. We assumed a NAAT cost of US$25, NAAT specificity of 99.6%, NAAT sensitivity of 100% for infants infected in pregnancy or at least 4 weeks prior to testing, and a mother-to-child transmission (MTCT) rate at 12 months of 4.9%; we simulated guideline-concordant rates of testing uptake, result return, and antiretroviral therapy (ART) initiation (100%). After diagnosis, infants were linked to and retained in care for 10 years (false-positive) or lifelong (true-positive). All parameters were varied widely in sensitivity analyses. Outcomes included number of infants with false-positive diagnoses linked to ART per 1,000 ART initiations, life expectancy (LE, in years) and per-person lifetime HIV-related healthcare costs. Both without and with confirmatory testing, LE was 26.2 years for HIV-infected infants and 61.4 years for all HIV-exposed infants; clinical outcomes for truly infected infants did not differ by strategy. Without confirmatory testing, 128/1,000 ART initiations were false-positive diagnoses; with confirmatory testing, 1/1,000 ART initiations were false-positive diagnoses. Because confirmatory testing averted costly HIV care and ART in truly HIV-uninfected infants, it was cost-saving: total cost US$1,790/infant tested, compared to US$1,830/infant tested without confirmatory testing. Confirmatory testing remained cost-saving unless NAAT cost exceeded US$400 or the HIV-uninfected status of infants incorrectly identified as infected was ascertained and ART stopped within 3 months of starting. Limitations include uncertainty in the data used in the model, which we examined with sensitivity and uncertainty analyses. We also excluded clinical harms to HIV-uninfected infants incorrectly treated with ART after false-positive diagnosis (e.g., medication toxicities); including these outcomes would further increase the value of confirmatory testing.

**Conclusions:**

Without confirmatory testing, in settings with MTCT rates similar to that of South Africa, more than 10% of infants who initiate ART may reflect false-positive diagnoses. Confirmatory testing prevents inappropriate HIV diagnosis, is cost-saving, and should be adopted in all EID programmes.

## Abstract 2

Lorna Dunning examine the cost-effectiveness of confirmatory testing in early infant HIV diagnosis programmes in South Africa.

## Author summary

**Why was this study done?:**

- Paediatric HIV remains a substantial burden in many low-resource countries, with 170,000 infants infected with HIV in 2015. The World Health Organization (WHO) recommends virological testing of HIV-exposed infants by 6 weeks of life, with immediate referral of those who test positive for initiation of HIV care to reduce infant mortality associated with HIV.
- Despite reported specificities greater than 99%, virological assays still have the possibility for false-positive diagnoses. The use of confirmatory testing is therefore strongly recommended in early infant diagnosis (EID) programmes.
- A recent policy survey by WHO demonstrated that implementation of confirmatory testing in EID programmes is limited, with 38% (8/21) of high-burden countries not including confirmatory testing for infants in their guidelines.
- Cost is often cited as a key barrier; many low-resource countries struggle to implement EID programmes due to the high costs of virological tests.
- Our study was designed to estimate the clinical and economic value of confirmatory testing in EID programmes.

**What did the researchers do and find?:**

- We used a computer simulation model of paediatric HIV infection, diagnosis, and treatment to examine the clinical and economic outcomes of EID programmes without and with confirmatory testing in South Africa.
- We found there was no clinical difference in the life expectancy or 1-year survival of HIV-infected infants between the 2 strategies, but without confirmatory testing, in settings with a mother-to-child transmission (MTCT) rate similar to that of South Africa, over 10% of infants initiating antiretroviral therapy (ART) would do so inappropriately as a result of a false-positive diagnosis.
- Confirmatory testing averted costly HIV care and ART in truly HIV-uninfected infants, leading the confirmatory testing strategy to be cost-saving compared to without confirmatory testing in all reasonable scenarios.
- ART should be initiated after the first positive test result; when ART was not initiated until the return of the confirmatory result, mortality during the delay to ART initiation substantially reduced survival for HIV-infected infants.

**What do these findings mean?:**

- Use of confirmatory testing in EID programmes, with ART initiation after the first positive result, substantially reduces the proportion of infants incorrectly diagnosed as HIV-infected and initiated on ART.
- Confirmatory testing is cost-saving under a wide range of scenarios.
- Confirmatory testing should be implemented in settings using virological assays for EID.

## Introduction

Despite the success of programmes to prevent mother-to-child transmission (MTCT) of HIV, paediatric HIV remains a substantial burden in sub-Saharan Africa, with 170,000 infants infected with HIV in 2015 [1]. Perinatally infected, untreated infants are at highest risk for rapid disease progression and mortality, with 1 in 2 untreated HIV-infected infants dying before their second birthday [2–5]. The World Health Organization (WHO) recommends testing HIV-exposed infants by 6 weeks of life and immediately referring those who test positive for initiation of HIV care and antiretroviral therapy (ART) [6].

Inexpensive serological antibody assays routinely used for diagnosis in adults cannot be easily interpreted for HIV-exposed infants (those born to HIV-infected women), because transplacental transfer of maternal antibodies leads infants to be seropositive for as long as 18 months [7,8]. Infants therefore require virological tests (nucleic acid amplification tests [NAATs] detecting HIV RNA or DNA) to diagnose HIV infection [9]. Despite reported specificities of >99%, NAATs still have the possibility for false-positive diagnoses [10,11]. As the incidence of paediatric HIV falls with improved access to ART for pregnant/breastfeeding women, the positive predictive value (PPV) of diagnostic assays also decreases, leading to a greater proportion of uninfected infants receiving false-positive diagnoses and starting ART. After ART is initiated, it may be impossible to distinguish truly infected infants from uninfected infants, because effective ART may lead to undetectable HIV RNA in HIV-infected infants whilst also preventing the development of endogenous anti-HIV antibody after maternal antibody fades from the circulation. Because truly uninfected infants may therefore have identical laboratory results to treated infected infants, they may face many years or even a lifetime of incorrect diagnosis and ART [7,12,13].

Current WHO and South African guidelines strongly recommend the use of confirmatory NAAT testing with ART initiation at the first positive result as part of early infant diagnosis (EID) programmes [6,14]. However, a recent policy survey by WHO demonstrated that despite confirmatory testing being used routinely for diagnosis of HIV in adults and across most domains of adult and paediatric medicine, the uptake of confirmatory testing within EID programmes remains limited, with 38% (8/21) of high-burden countries not including confirmatory testing for infants in their guidelines [15–17]. Even where guidelines recommend confirmatory testing, it is rarely implemented [18]. Cost is often cited as a key barrier; many low-resource countries struggle to implement EID programmes due to the high costs of the NAATs required for infant diagnosis. We used a computer simulation model of paediatric HIV infection, diagnosis, and treatment to examine the clinical and economic outcomes of EID programmes without and with confirmatory testing in South Africa.

## Methods

### Overview

We used the Cost-Effectiveness of Preventing AIDS Complications (CEPAC)–Pediatric model to simulate a cohort of HIV-exposed infants in South Africa undergoing 2 EID algorithms: 6-week EID testing without confirmatory testing and 6-week EID testing with confirmatory testing. The CEPAC–Pediatric model is a first-order Monte Carlo simulation model of paediatric HIV infection, disease progression, diagnosis, and treatment [19–21]. For this analysis, we simulated HIV-exposed infants (born to women living with HIV) from birth through death. Risk of intrauterine or intrapartum HIV infection was modelled as a 1-time risk, based on 3 key maternal characteristics: the probability a mother was aware of her HIV diagnosis during pregnancy; the probability that she received ART during pregnancy, reflecting prevention of MTCT (PMTCT) coverage; and maternal CD4 count for women not receiving ART, reflecting disease stage. Uninfected infants faced a monthly risk of postpartum transmission based on these same characteristics until complete cessation of breastfeeding. All simulated patients faced monthly risks of non-HIV-related mortality. After HIV infection occurred, patients faced additional risks of opportunistic infections (OIs) and HIV-related mortality based on their age, CD4 percent (age < 5 years) or CD4 count (age ≥ 5 years), retention in care, and ART use. Full details of the CEPAC–Pediatric model structure are available in S1 Text and S2 Table and at http://web2.research.partners.org/cepac/model.html.

This work was approved by the Partners Human Research Committee, Boston, MA, US.

### Modelled population

In the base-case analysis, we simulated HIV-exposed South African children presenting to care at 6 weeks of age. EID in South Africa is currently directed at infants with known HIV exposure [14]; we therefore included only infants born to women identified as living with HIV during pregnancy. South African guidelines currently recommend ‘Option B+’, or lifelong ART for all pregnant/breastfeeding women identified as living with HIV [14]. We assumed that 90% of women had access to ART during pregnancy and breastfeeding [1]. Based on early data after the release of new infant feeding guidelines in South Africa, we assumed 80% of the cohort was breastfed, for a mean duration of 12 months [6,14,22].

### Model outcomes

The model records true infection status for all infants, as well as the results of each administered assay, allowing the direct reporting of true-positive, true-negative, false-positive, and false-negative diagnoses. The primary model outcomes were the number of infants with false-positive diagnoses linked to care as a proportion of total ART initiations, PPV (defined as the proportion of positive test results due to truly HIV-infected infants), life expectancy (LE, in years), and average per-person lifetime HIV-related healthcare costs, from the perspective of the healthcare provider. We projected survival, LE, and costs separately for HIV-infected infants and for the complete birth cohort of HIV-exposed infants, which included both HIV-infected and HIV-uninfected infants. Costs are presented in 2013 US dollars; costs and life expectancies were modelled both undiscounted and discounted at a rate of 3%/year [23]. We first calculated per-person outcomes, then translated these to population outcomes for all 350,000 HIV-exposed infants born in South Africa in 1 year [24,25]. Where clinical outcomes were equal for both strategies, calculation of incremental cost-effectiveness was not necessary; we considered alternative strategies as either cost-saving or more costly in these cases.

### Modelled strategies

The strategy ‘without confirmatory testing’ simulated all HIV-exposed infants receiving a NAAT at 6 weeks of age; in this strategy, all infants who received positive results initiated ART upon result return (mean turnaround time: 1 month). The strategy ‘with confirmatory testing’ simulated all HIV-exposed infants receiving a NAAT at 6 weeks of age; all infants who received positive results on the first test initiated ART at result return, but a second blood sample was drawn before ART initiation and sent for a confirmatory NAAT [6,14]. We assumed conditional independence of the primary and confirmatory NAAT results, because WHO recommends that a second specimen be used for confirmatory testing, and most false-positive NAAT results are likely consequences of specimen handling error rather than biological phenomena [6,26,27]. Infants with negative confirmatory tests underwent a third test per WHO guidelines before HIV infection was ruled out and ART stopped. For infants who were truly uninfected but initiated ART and therefore entered HIV care after a false-positive diagnosis (both strategies), we assigned costs for 10 years of routine HIV care, ART, and laboratory monitoring. We varied this duration widely in sensitivity analysis. For truly infected infants diagnosed and linked to care, the model includes lifetime costs for routine care, ART, and laboratory monitoring, as well as care for OIs. We conservatively excluded clinical harms from incorrect ART initiations, such as medication toxicity and stigma.

### Input data

We modelled cohort characteristics, PMTCT coverage and MTCT risks, disease progression, and ART outcomes using data from published trials and cohort studies in sub-Saharan Africa (Table 1; Table A in S1 Text; S2 Table) [28–32]. The specificity of NAATs was modelled as 99.6%, from a 2015 WHO meta-analysis [33]. We modelled NAAT sensitivity as a function of time since infection, reaching 100% among infants infected during pregnancy or at least 4 weeks prior to testing (Table 1) [34]. In the base case, laboratory-based NAAT cost was US$25, which included assays, reagents, and human personnel resources associated with specimen processing and result return. To describe the full potential impact of each strategy, in the base-case analysis we modelled guideline-concordant (100%) probabilities of presentation to testing, result return (first NAAT: 1 month), and ART initiation after an HIV diagnosis; we varied these widely in sensitivity and scenario analyses to reflect implementation across a range of settings [1,35–37].

**Table 1:** Selected data parameters for CEPAC–Pediatric model analysis of early infant HIV diagnosis testing in South Africa.

Parameter	Subcategory	Base-case value	Range examined	Source
**Cohort characteristics**				
Age, months (SD)		0 (0)	—	Assumption
Percent male infants		48.8%	—	[28]
Mothers with CD4 ≤ 350 cells/μl before ART		36%	30–50	[44]
Breastfeeding (proportion of all infants)		80%^a^	50–100	[45]
Mean breastfeeding duration, months (SD)		12 (2)	3–18	Assumption
**Mother-to-child transmission parameters, percent**				
*Intrauterine/intrapartum—1-time risk*				
On ART (60% IU transmission; 40% IP transmission)	Maternal CD4 ≤ 350 cells/μl	1.0	×0.5–2.0	[46–49]
	Maternal CD4 > 350 cells/μl	1.0	×0.5–2.0	
Not on ART (60% IU transmission; 40% IP transmission)	Maternal CD4 ≤ 350 cells/μl	27	×0.5–2.0	[50–55]
	Maternal CD4 > 350 cells/μl	17	×0.5–2.0	
*Postpartum)—monthly risk during breastfeeding*				
On ART	Maternal CD4 ≤ 350 cells/μl	0.19	×0.5–2.0	[56–61]
	Maternal CD4 > 350 cells/μl	0.19	×0.5–2.0	
Not on ART—exclusive breastfeeding	Maternal CD4 ≤ 350 cells/μl	0.76	×0.5–2.0	[44,50,62,63]
	Maternal CD4 > 350 cells/μl	0.24	×0.5–2.0	
Not on ART—mixed or complementary feeding	Maternal CD4 ≤ 350 cells/μl	1.28	×0.5–2.0	[44,50,62,63]
	Maternal CD4 > 350 cells/μl	0.40	×0.5–2.0	
**PMTCT cascade parameters, percent**				
Probability maternal status known in pregnancy		100%	—	Assumption
Probability mother on ART in pregnancy and breastfeeding		90%	40–100	[1]
Monthly maternal mortality risk	Maternal CD4 ≤ 350 cells/μl	0.21	×0.5–2.0	CEPAC adult model
	Maternal CD4 > 350 cells/μl	0.11	×0.5–2.0	
**EID cascade parameters, percent guideline compliant**				Scenario-specific assumptions
Probability of presenting to a testing visit		100%	0–100	
Probability of being offered and accepting test		100%	0–100	
Probability of receiving test results		100%	0–100	
Delay between primary test and result receipt, months (SD)	Standard NAAT	1 (0)	0–5	
	POC NAAT	0 (0)	0–5	
Probability of linking to care/ART after diagnosis		100%	0–100	
**NAAT assay characteristics, base-case percent**				
Sensitivity of standard NAAT for IU infection	All ages	100%	90–100	[33,64]
Sensitivity of standard NAAT for IP infection	Month 1	0%	—	[33,64]
	Later months	100%	90–100	
Sensitivity of standard NAAT for PP infection (by time since infection)	Month of infection	0%	—	[33,64]
	Later months	100%	90–100	
Specificity of standard NAAT	All ages	99.6%	90–100	[33,64]
Sensitivity of POC	All ages	95.5%	90–100	[65,66]
Specificity of POC	All ages	99.8%	—	[65,66]
**ART outcomes, percent**				
*ART efficacy*: *HIV RNA < 400 copies/ml at 24 weeks on ART*				
Ages 0–59 months	First-line ART^b^	91%		[29,30]
	Second-line ART^c^	75%		
Ages 60+ months	First-line ART^b^	75%		[67]
	Second-line ART^c^	75%		
*ART-associated CD4-independent risk reductions*				
Risk reduction in OI (age 0–12)		85%		[20]
Risk reduction in OI (age 13+)		32%		[68]
Risk reduction in mortality (age 0–12)		90%		[20]
Risk reduction in mortality (age 13+, range by CD4)		55%–96%		[68]
Monthly loss to follow-up after ART initiation		0.2%		[29,30]
**Costs, US dollars**				
OI care (per event, range by age, CD4%/CD4, type of event)		260–2,175	×0.5–2.0	[39,42,43,69]
Major toxicity event		1,972	×0.5–2.0	[39]
ART (per month, range by regimen, dose/age)		7–40	×0.5–2.0	[42,43]
NAAT	Standard (laboratory)	25	10–400	Assumption
	POC	30	30–50	
Negative NAAT result return		1.83		Assumption (nurse time × salary) [70]
Positive NAAT result return		3.05		
Routine HIV care (per month, range by age)		20–165	×0.5–2.0	[39]

A full description of all model input parameters, as well as ranges for sensitivity analyses and uncertainty analyses, is provided in Tables A and B in S1 Text.

^a^Of the total population of breastfed infants (80% in the base case), for the first 6 months of life: exclusive breastfeeding in 55%; mixed breastfeeding in 25%; replacement feeding from birth in 20%. After 6 months of age, all infants still breastfeeding are assumed to have complementary feeding (breastmilk and other liquids/solids).

^b^Lopinavir, ritonavir, abacavir, and lamivudine.

^c^Efavirenz plus zidovudine and lamivudine.

ART, antiretroviral therapy; CEPAC, Cost-effectiveness of Preventing AIDS Complications; EID, early infant diagnosis; IP, intrapartum; IU, intrauterine; NAAT, nucleic acid amplification test; OI, opportunistic infection; PMTCT, prevention of mother-to-child transmission; POC, point-of-care; PP, postpartum; SD, standard deviation.

We incorporated published South African healthcare costs for HIV-infected children less than 5 years of age. Costs associated with OI care for children 5 years of age and older were calculated from South African adult resource utilisation (outpatient visits, inpatient days, and laboratory testing) multiplied by South African unit costs [38,39]. Routine HIV care costs (all ages) ranged by age from US$20 to US$165 per month [40,41]. First-line ART costs were from Clinton Health Access Initiative price lists and WHO weight-based dosing recommendations, ranging by age and weight from US$7 to US$40 per month (Table 1) [42,43].

### Sensitivity analyses

Following ISPOR–SMDM Good Research Practices Task Force (S1 Table) guidelines on uncertainty in model-based analyses, we conducted extensive univariate and multivariate sensitivity analyses (Tables B and D in S1 Text) [71]. We first conducted univariate sensitivity analyses, in which we varied NAAT specificity and sensitivity, infant HIV prevalence via PMTCT coverage and MTCT risks, the probability of initiating ART after a positive NAAT result, the amount of time infants with a false-positive diagnosis spent on ART, the costs of NAATs, routine HIV care and OI care costs, and ART treatment costs (S1 Table; Task Force recommendation VI-9). Holding all other parameters at their base-case values, we identified the threshold value for each parameter at which the comparison between with and without confirmatory testing would change (S1 Table; Task Force recommendation VI-12). We next performed multivariate sensitivity analyses to evaluate the impact of simultaneous variation in multiple parameters (S1 Table; Task Force recommendation VI-10). We also examined the assumption of conditional independence of the first and confirmatory assays by varying their specificities simultaneously, and assigned a combination of assay cost, sensitivity, specificity, result return time, and result return probability to reflect emerging point-of-care (POC) EID assays (Table 1).

### Implementation scenario analyses

We conducted 3 scenario analyses to examine important issues in EID implementation (S1 Table; Task Force recommendation VI-10). First, we simulated lower rates of implementation of 6-week EID testing incorporating input parameters from current testing and treatment cascades in resource-limited countries. We modelled (a) probability of presenting to testing as 73% and probability of ART initiation as 71% of infants with a positive test result and (b) probability of presenting to testing as 95% (2016 UNAIDS estimates for EID coverage in South Africa), probability of result return to caregiver as 80%, and probability of ART initiation as 71% [1,35–37]. Second, we examined programmes offering routine EID testing at both birth and 6 weeks of age to all HIV-exposed infants. Birth tests were modelled as standard laboratory-based NAAT tests with a 1-month result return time (Table 1). Third, WHO strongly recommends prompt ART initiation after a first positive test result (as in our base-case analysis) to avoid delays during a period of high mortality without treatment, but that NAAT testing be repeated on a second specimen drawn prior to ART initiation, to confirm the initial diagnosis [6,7]. Because some providers may be reluctant to initiate ART before receiving a confirmatory result, we also examined the impact of postponing ART until return of the confirmatory test result (1–2 months).

### Uncertainty analyses

The univariate sensitivity analyses described above reveal the sensitivity of policy conclusions to variations in key parameters through a wide range of plausible values. To reflect the uncertainty in the primary data estimates, we also varied key parameters through reported 95% confidence intervals (or range or interquartile ranges, if 95% confidence intervals were not available; S1 Table; Task Force recommendations VI-6 and VI-7). For most parameters, this interval fell well within the range examined in sensitivity analyses. We used the results of previously reported model calibration and validation analyses [19,20] to examine the impact of parameter and model structural uncertainty on the comparison between without and with confirmatory testing (S1 Table; Task Force recommendation VI-14).

## Results

### Base-case results: Clinical outcomes

In the base-case analysis, the birth cohort was projected to include 1.8% infants with intrauterine HIV infection, 1.2% with intrapartum infection, and 1.9% with postpartum infection (cumulative MTCT risk 4.9%), with 95.1% of the cohort HIV-exposed but uninfected (Table 2). In both 6-week EID algorithms (without and with confirmatory testing), HIV-infected infants had a projected 1-year survival of 75.7% and LE of 26.2 years; there was also no clinical difference between the 2 strategies for the entire birth cohort (1-year survival 93.3%, LE 61.4 years). Without confirmatory testing, 128 infants of every 1,000 who initiated ART were truly uninfected, reflecting false-positive diagnosis; this led to a PPV of 87.2%. With confirmatory testing, this proportion fell to only 1 in 1,000 ART initiations, for a PPV of 99.9%.

**Table 2:** Base-case model results: Early infant HIV diagnosis testing at 6 weeks in South Africa with and without confirmatory testing.

**a. Clinical outcomes**				
	**HIV infected infants**		**Birth cohort**	
**MTCT outcomes**				
	4.9% of entire birth cohort: 1.8% IU, 1.2% IP, 1.9% PP		4.9% HIV-infected 95.1% HIV-exposed uninfected	
**Clinical outcomes**^**a**^				
1-year survival	75.7%		93.3%	
Life expectancy (years, undiscounted)	26.2		61.4	
**b.Economic outcomes**				
**EID strategy**	**Number of infants with false-positive diagnoses initiating ART per 1,000 ART initiations**	**Lifetime cost per HIV-exposed infant (US dollars**^**b**^	**Proportion of total lifetime costs due to care for infants with false-positive diagnoses**	**Positive predictive value of assay**
*6-week EID without confirmatory testing*	128	$1,830	2.1%	87.2%
*6-week EID with confirmatory testing*	1	$1,790	0.01%	99.9%

^a^Results are shown for both strategies. We simulate ART initiation after the first positive EID assay result is received, with ART cessation if a confirmatory assay is subsequently negative. Because HIV-infected infants do not delay ART initiation for a confirmatory test result, the projected life expectancy for both EID strategies is equal.

^b^Costs are in 2013 US dollars and are undiscounted.

ART, antiretroviral therapy; EID, early infant diagnosis; IP, intrapartum; IU, intrauterine; MTCT, mother-to-child transmission; PP, postpartum.

### Base-case results: Costs and cost-savings

**Fig 1:**
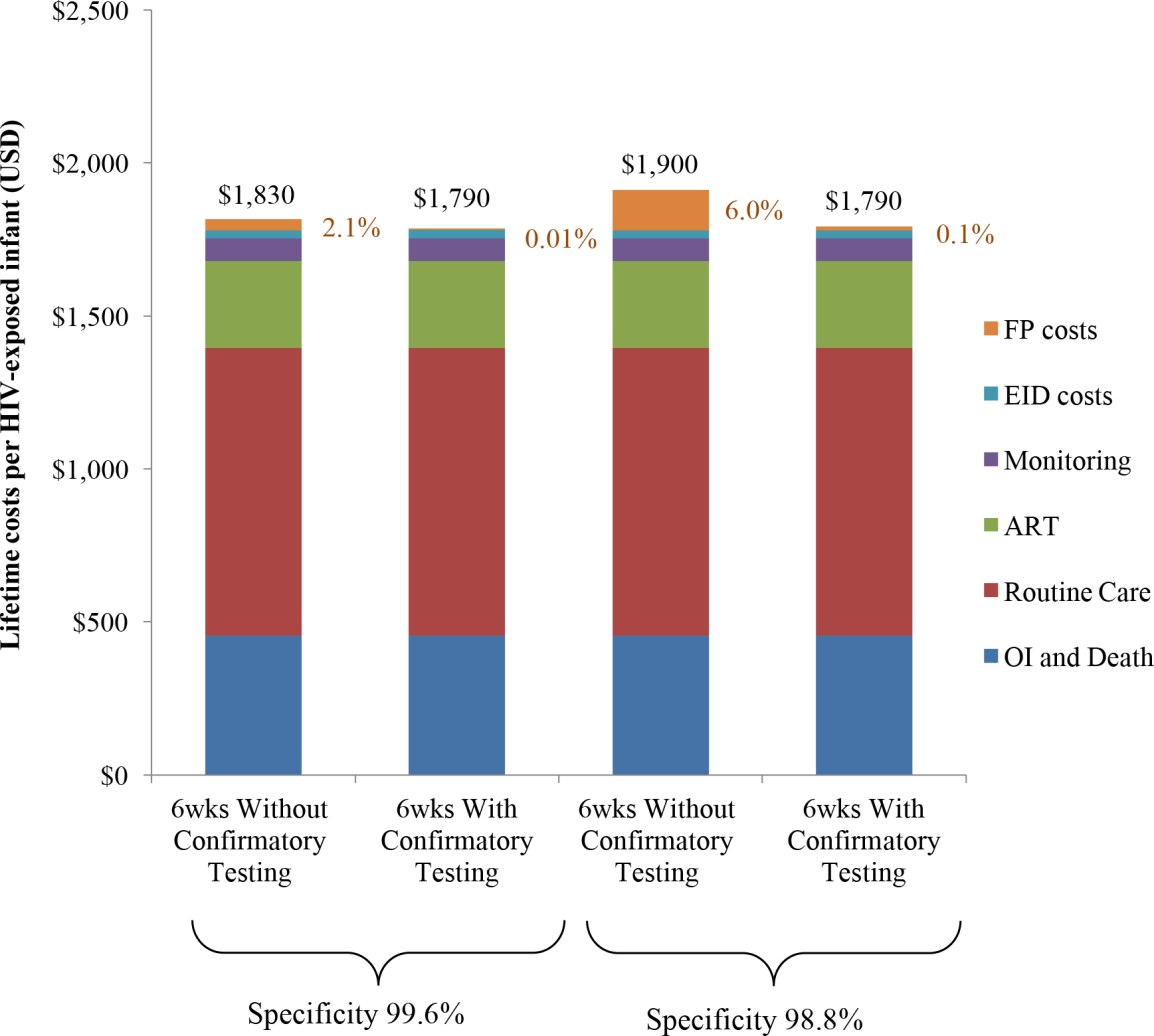
Total lifetime costs per HIV-exposed infant by EID strategy. Columns include components of lifetime total costs per HIV-exposed infant tested: routine HIV care, CD4 and HIV viral load monitoring, OIs and end-of-life care, ART, EID costs, and false-positive costs. EID programme costs are shown in blue and comprise 2%–3% of lifetime costs, as shown previously [21]; false-positive costs are shown in orange and are made up of all component costs acquired for HIV-infected infants other than OI costs. ART, antiretroviral therapy; EID, early infant diagnosis; FP, false-positive; OI, opportunistic infection.

Lifetime costs of HIV-exposed infants were US$1,830/infant without confirmatory testing and US$1,790/infant with confirmatory testing (Table 2; Fig 1). The approach of using confirmatory testing was therefore equally effective and was cost-saving; it became cost-saving 3 months after ART initiation (Fig A in S1 Text). Without confirmatory testing, 2.1% of total lifetime HIV-related costs for the birth cohort were accrued by truly uninfected infants after false-positive diagnoses, compared to only 0.01% of lifetime costs with confirmatory testing (Fig 1, orange). If 6-week EID programmes were available for the 350,000 HIV-exposed infants born annually in South Africa, 11,000 infants would require a second NAAT to confirm a first positive result. The cost of these confirmatory NAATs would be approximately US$260,000, but they would avert unnecessary HIV care and ART for 1,400 infants. By averting unnecessary ART and HIV costs for uninfected infants, confirmatory testing would save over US$1,050,000 in the first year and US$13,860,000 in lifetime costs for a South African birth cohort, compared to the approach of not using confirmatory testing [24,25].

### Univariate sensitivity analyses: Costs

**Fig 2:**
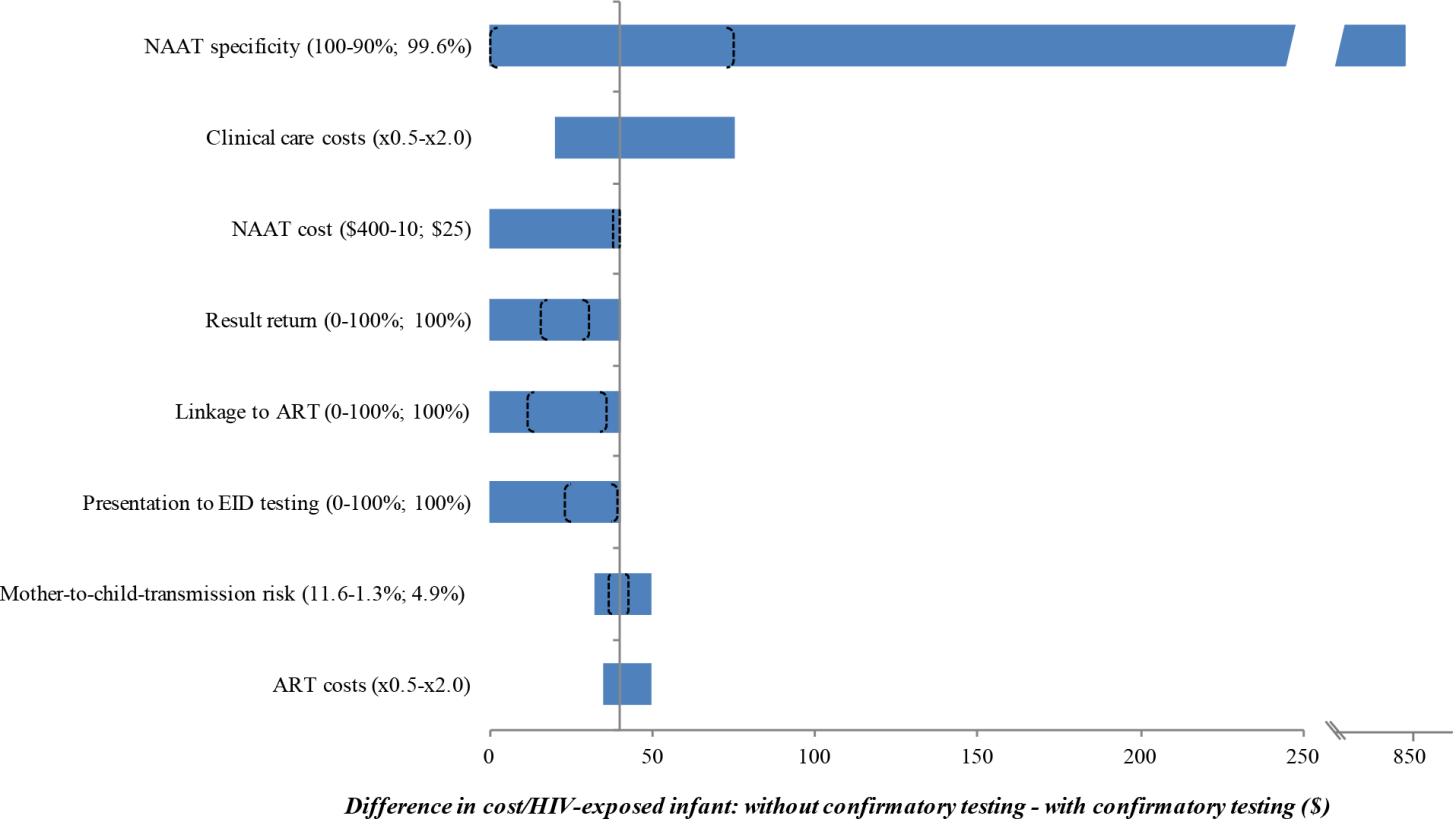
Univariate sensitivity analyses examining the impact of variation in individual input parameters on the difference in cost per HIV-exposed infant between the without and with confirmatory testing strategies. Key parameters varied in sensitivity analyses are shown on the left. Values in parentheses indicate the range examined (from the value leading to the lowest difference in cost to the value leading to the greatest difference, with base-case values after the semicolon). The horizontal axis shows the difference in cost between the 2 strategies: without confirmatory testing minus with confirmatory testing. The bounds of the blue bar indicate the cost differences at the extreme parameter values; longer bars therefore indicate parameters to which the model results were more sensitive. Where confidence intervals were available for the primary data estimates used in the base case, we indicate the bounds of these confidence intervals with brackets overlying the blue bars; the distance between brackets therefore indicates the degree to which the base-case estimates are affected by parameter uncertainty. The blue bar reaches the far left axis (indicating a cost difference of 0) at the threshold value for each parameter where confirmatory testing is no longer cost-saving compared to without confirmatory testing. The grey vertical line indicates the value for each parameter at the base-case result: a savings of US$40 per infant with confirmatory testing. ART, antiretroviral therapy; EID, early infant diagnosis; NAAT, nucleic acid amplification test.

The use of confirmatory testing remained cost-saving even with wide variations in model parameters, including the cost, specificity, and sensitivity of the NAAT; the probability of presentation at each step in the EID cascade; and the costs of routine care and OI care. Robustness of model results to these key parameters is shown in Fig 2. However, there were 3 key exceptions: confirmatory testing was no longer cost-saving if the duration spent by HIV-uninfected infants in care and on ART after false-positive diagnosis was <3 months (Fig A in S1 Text), if the first and confirmatory assays were no longer considered to be independent (specificity of confirmatory assay <15%; Tables D and E in S1 Text), or if the cost of the NAAT was 16-fold higher than in the base case (>US$400).

We repeated the base-case analysis using a NAAT specificity of 98.8%, as reported by WHO in a previous meta-analysis [33]. The cost-savings associated with confirmatory testing were greater than in the base case (US$1,900/infant without confirmatory testing; US$1,790/infant with confirmatory testing; Fig 1; Table C in S1 Text). With this lower specificity, false-positive diagnoses accounted for 6.0% of total lifetime costs without confirmatory testing, compared to 0.1% with confirmatory testing. After false-positive diagnosis in this scenario, if the HIV status of infants incorrectly identified as infected could be ascertained and their ART interrupted within 2 months of starting ART, confirmatory testing would no longer be cost-saving.

### Univariate sensitivity analyses: Clinical outcomes

**Fig 3:**
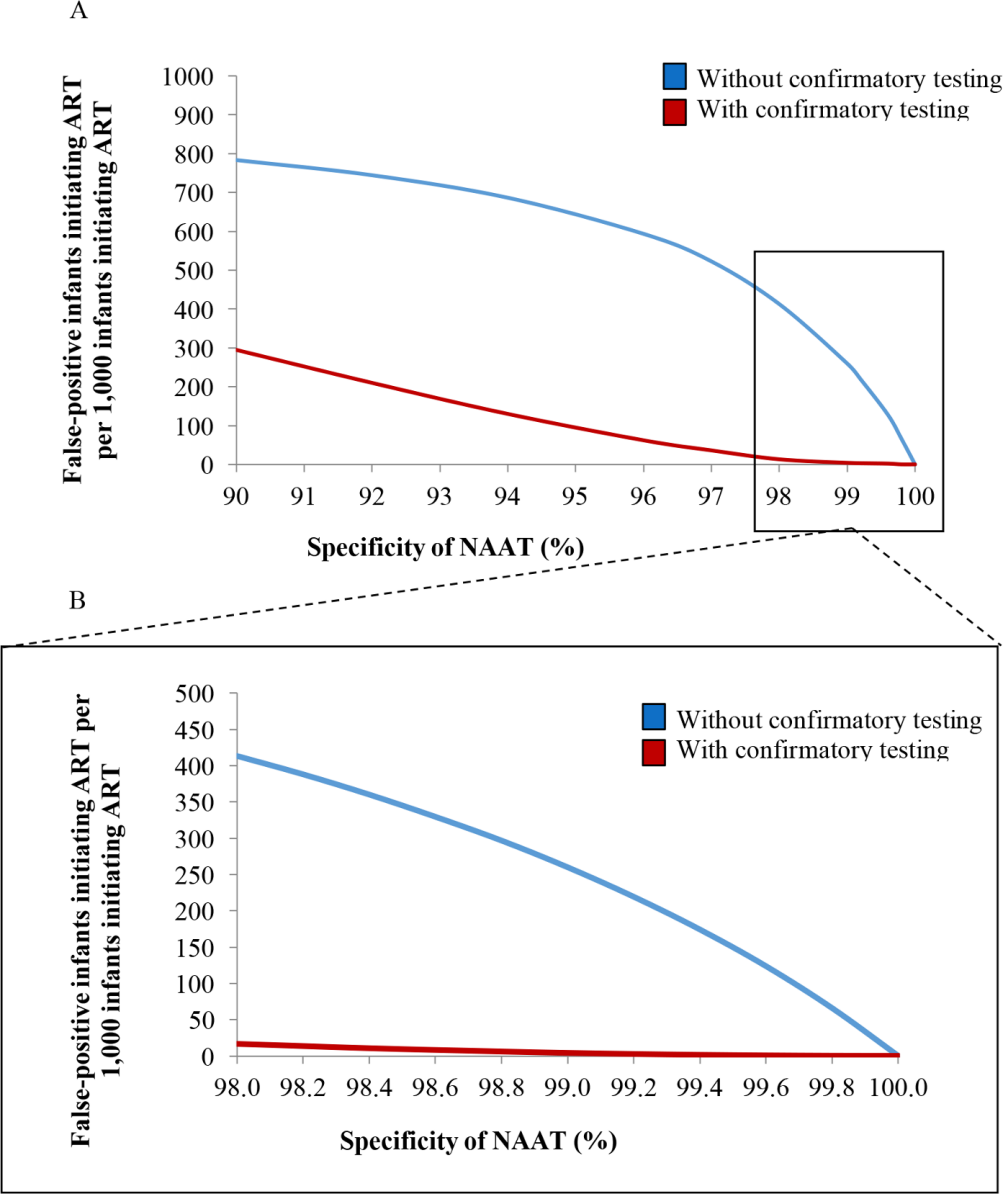
Number of infants linked to ART after false-positive diagnosis, per 1,000 ART initiations, by assay specificity. (A) Univariate sensitivity analysis varying NAAT specificity without and with confirmatory testing for 6-week EID testing. The vertical axis depicts the number of infants with a false-positive diagnosis who initiate ART, per 1,000 ART initiations. The horizontal axis depicts the specificity of the NAAT. (B) The inset panel depicts results at higher specificity values, as reported for most NAATs (Table 1). ART, antiretroviral therapy; EID, early infant diagnosis; NAAT, nucleic acid amplification test.

Several variations in model parameters changed the proportion of ART initiations due to false-positive diagnosis (Table D in S1 Text). Increases in specificity, such as those recently described for novel POC EID assays, reduced the number of false-positive diagnoses in the approach without confirmatory testing to 69/1,000 ART initiations (Table D in S1 Text; Fig 3) [65,66]. A reduction in NAAT sensitivity of 5% only minimally increased the number of infants initiating ART after false-positive diagnosis without confirmatory testing (from 128/1,000 ART initiations in the base case to 133/1,000), but LE for HIV-infected infants was reduced to 25.9 years without confirmatory testing and to 25.7 years with confirmatory testing (Table D footnote in S1 Text). Reductions in the number of infected infants successfully undergoing EID testing, receiving test results, and initiating ART had minimal effects on the proportion of ART initiations due to false-positive diagnoses, but reduced the LE for HIV-infected infants: if only 50% of infected infants completed the EID cascade, LE for HIV-infected infants was reduced to 23.6 years (Table D in S1 Text).

### Multivariate sensitivity analyses

**Fig 4:**
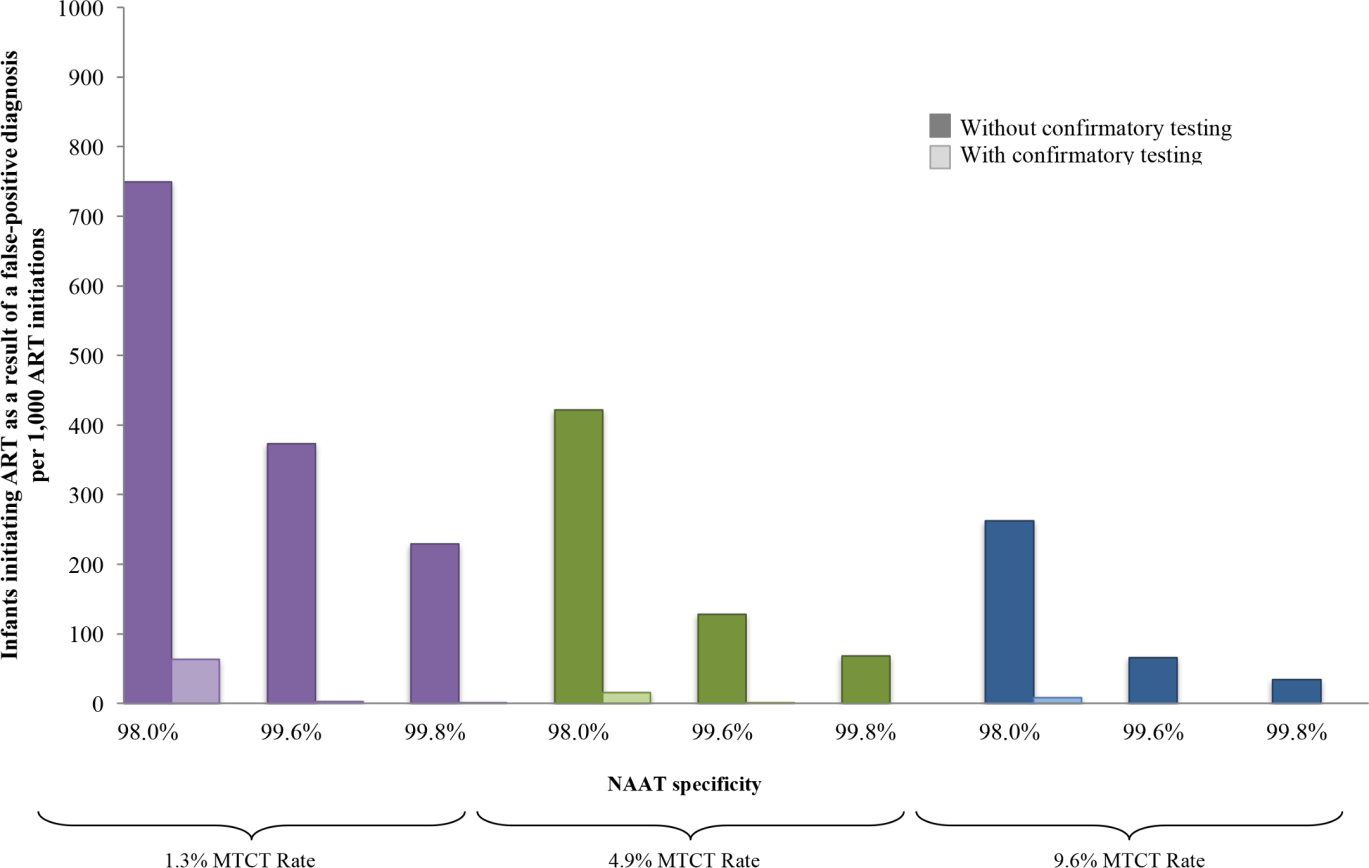
Number of infants linked to ART after false-positive diagnosis, per 1,000 ART initiations, by assay specificity and MTCT risk. Multivariate sensitivity analysis varying specificity of the NAAT and infant HIV prevalence modelled by increasing MTCT risk. The vertical axis shows the number of infants with false-positive diagnosis initiating ART, per 1,000 ART initiations. Groups of coloured bars indicate 3 values for infant HIV prevalence at weaning (12 months of age): purple indicates a low MTCT risk scenario, with 12-month risk of 1.3%; green indicates the base-case value of 4.9%; and blue indicates a high MTCT risk scenario, with risk of 9.6%. Three values of NAAT specificity are shown within each MTCT risk scenario. For each combination of MTCT risk and NAAT specificity, bars indicate those who are truly HIV-uninfected (false-positive diagnosis). The left, dark-coloured bar in each pair reflects the outcome without confirmatory testing, and the right, light-coloured bar reflects the outcome with confirmatory testing. ART, antiretroviral therapy; EID, early infant diagnosis; MTCT, mother-to-child transmission; NAAT, nucleic acid amplification test.

In multivariate sensitivity analyses, results were also sensitive to reductions in NAAT specificity and infant HIV prevalence (lower MTCT risks), as depicted in Fig 4. When MTCT risks were <1.3% and NAAT specificity was 98.0%, 749/1,000 infants initiating ART without confirmatory testing were truly uninfected (Fig 4; Table F in S1 Text). With a very high MTCT risk of 9.6% and NAAT specificity of 99.8%, this fell to 34/1,000 ART initiations without confirmatory testing. In all multivariate sensitivity analyses varying specificity (<100%) and infant HIV prevalence, confirmatory testing remained cost-saving (Table F in S1 Text).

### Implementation scenario analyses

Incorporating input parameters from current testing and treatment cascades reduced the LE of HIV-infected infants, but the number of infants with a false-positive diagnosis initiating ART remained 128/1,000 ART initiations (Table 3). For programmes offering EID at both birth and 6 weeks of age, the proportion of infants initiating ART after false-positive diagnosis without confirmatory testing increased to 213/1,000 ART initiations, accounting for 4.0% of total costs (Table D and Fig B in S1 Text). When ART was not initiated until the return of the confirmatory NAAT result, mortality during the delay to ART initiation reduced the projected 1-year survival (74.8%) and LE (25.9 years) for HIV-infected infants with confirmatory testing; results for the approach without confirmatory testing (1-year survival 75.7%; LE 26.2 years) were unchanged (Table D in S1 Text). HIV-related healthcare costs remained lower with confirmatory testing than without confirmatory testing in all 3 scenarios.

**Table 3:** Implementation scenario model results: Early infant HIV diagnosis testing at 6 weeks in South Africa with and without confirmatory testing.

EID strategy	Life expectancy (HIV-infected infants, years)	Number of infants with false-positive diagnoses initiating ART per 1,000 ART initiations	Proportion of total lifetime costs due to care for infants with false-positive diagnoses	Lifetime cost per HIV-exposed infant (US dollars)
**95% presentation to EID, 100% result return, 71% ART initiation**				
With confirmatory testing	24.6	1	0.006%	$1,685
Without confirmatory testing	24.6	128	1.5%	$1,710
**95% presentation to EID, 80% result return, 71% ART initiation**				
With confirmatory testing	23.8	1	0.004%	$1,630
Without confirmatory testing	23.8	128	1.2%	$1,650
**73% presentation to EID, 100% result return, 71% ART initiation**				
With confirmatory testing	23.7	1	0.005%	$1,620
Without confirmatory testing	23.7	128	1.2%	$1,640

ART, antiretroviral therapy; EID, early infant diagnosis.

### Uncertainty analyses

The impact of varying key parameters through the confidence intervals around base-case estimates is shown in Fig 2. In all parameter sets derived from formal calibration analyses, confirmatory testing remained cost-saving (Fig C in S1 Text).

## Discussion

We simulated EID strategies without and with confirmatory testing for HIV-exposed infants in South Africa. The primary finding was that, when confirmatory testing was not used in the model, more than 10% of infants who initiated ART reflected false-positive diagnoses. These model results are comparable to empirical data from Africa: when records of infants receiving positive EID results and/or initiating ART were reviewed in detail, the proportion found to be truly HIV-uninfected was 2.5% in Kenya, 6.3% in South Africa, 14.6% in Malawi, and 16% in Côte d’Ivoire and Burkina Faso [72–75]. HIV-uninfected infants incorrectly initiating ART not only receive unnecessary medication exposure and treatment costs, but may also experience long-term medication toxicities and the substantial stigma associated with HIV diagnosis [7]. To remain conservative, we did not include these detriments in our analysis. Confirmatory testing would therefore prevent a broader set of adverse outcomes than those described here in a substantial proportion of HIV-uninfected infants.

False-positive results occur for a variety of reasons but are likely the result of stretched human resources compromising test specificity and sensitivity via specimen handling errors such as mislabelling of specimens, incorrect specimen placement in the device, or inadequate quality control resulting in contamination between specimens during PCR amplification [27,76]. WHO recommends the use of a second specimen for a confirmatory test, ensuring the independence of the 2 assays and reducing the likelihood of a second false-positive result. Ensuring that an initial HIV diagnosis is correct is essential because identifying a misdiagnosed, truly HIV-uninfected infant remains extremely difficult after ART is initiated [6]. Lack of detectable anti-HIV antibody, HIV RNA, or HIV DNA may reflect either absence of true infection or the impact of effective ART, which makes withdrawal of treatment the only mechanism available for clinicians wishing to determine the true HIV status of an infant on ART. However, treatment interruptions are not currently recommended, due to concerns about viral rebound and disease progression [77,78]. Unconfirmed EID test results therefore cause diagnostic dilemmas for the provider, whilst families may experience confusion and uncertainty about the health system, discouraging future engagement in care [79].

The second key finding from this analysis is that including confirmatory testing in EID programmes is cost-saving. We found that confirmatory testing reduced the cost per HIV-exposed infant tested from US$1,830 to US$1,790. Although concerns have been raised about the capacity of laboratories to conduct the additional assays needed for confirmatory testing, we found that the number of additional tests was relatively small, as they are required by only 3% of HIV-exposed infants [9]. The costs of these additional tests to confirm HIV infection would be offset by the reduction in false-positive diagnoses and their associated unnecessary HIV care, ART, and ART toxicity costs. Infants with a false-positive diagnosis only had to remain in care and on ART for longer than 3 months for confirmatory testing to become cost-saving; a shorter duration on ART would be extremely unlikely due to the difficulty of identifying uninfected infants after ART initiation. Confirmatory testing remained cost-saving despite wide variations in the costs of clinical care and ART, and at any plausible NAAT cost. In addition, our findings demonstrate that confirmatory testing is cost-saving even with increases in the probability of becoming lost to follow-up between presenting to EID testing, receiving test results, and initiating ART, but lowering the number of HIV-infected infants initiating ART reduces LE for truly infected infants.

A third key finding of this analysis is that when confirmatory testing is done, ART should be initiated after the first positive result, as WHO recommends. If ART was delayed until the result of the confirmatory assay was available, short-term survival and LE were markedly reduced. Waiting even 1 month to initiate ART until the return of the confirmatory result can reduce 1-year survival for HIV-infected infants substantially (from 75.8% to 74.5%) and overall LE for HIV-infected infants (from 26.2 years to 25.9 years). Novel POC EID assays, which offer in-clinic testing and same-day result return, have been proposed as an approach to improving timely ART initiation. Based on preliminary published values for POC NAAT sensitivity (95.5%), specificity (99.8%), and cost (US$30), our analyses suggest that confirmatory testing would likely remain cost-saving in EID programmes using POC assays in place of traditional laboratory assays [65,66].

In South Africa, access to ART during pregnancy has steadily increased, leading to a 76% reduction in new HIV infections among children [1]. If PMTCT programmes continue their success, MTCT rates will fall below 2%, as they have in countries such as Thailand and Cuba [80,81]. As expected, we found that confirmatory testing becomes increasingly critical to reduce false-positive diagnoses when MTCT risks are very low, due to the reduced PPV of diagnostic assays at low disease prevalence (Fig 3). This remained true even when NAAT specificity was extremely high (99.9%). In addition to disease prevalence, assay sensitivity also contributes to PPV. We found that small reductions in NAAT sensitivity, a theoretical result of maternal and infant ART for treatment and MTCT prevention [82], would only minimally increase the number of infants initiating ART after false-positive diagnosis, although lower sensitivity would lead to fewer infected infants being identified and would reduce LE for HIV-infected infants. Finally, opportunities for false-positive results increase when testing is done twice; the inclusion of confirmatory testing within the EID cascade is even more important when programmes consider the addition of birth testing to a 6-week testing programme.

There were 3 main limitations of the analysis. First, uncertainty exists in all long-term projections; although our model was calibrated to ensure results match current survival and OI data, developments in treatment and technology will undoubtedly occur in the coming years [19,20]. Our model structure does not currently permit formal probabilistic sensitivity analyses; however, we performed extensive univariate and multivariate sensitivity analyses to assess the impact of uncertainty around all key input parameters, in keeping with international guidelines [71] (Table G in S1 Text). Confirmatory testing remained cost-saving in a wide range of evaluated scenarios, unless the unlikely thresholds shown in Fig 2 were met or surpassed. Second, while we included all relevant costs for HIV-uninfected infants on ART, we did not include negative clinical impacts for these patients, such as stigma, morbidity or mortality related to ART toxicity, or reduced quality of life after false-positive diagnosis. Including such harms from false-positive diagnosis would further improve the value of confirmatory testing. Finally, this work primarily addresses HIV-exposed infants in South Africa, although our findings may be generalisable to a range of settings: sensitivity analyses demonstrated that confirmatory testing remained cost-saving even with wide variations in MTCT risk, NAAT specificity and sensitivity, NAAT costs, and costs for HIV care and ART.

In summary, we find that use of a second NAAT for confirmatory testing in EID programmes substantially reduces the proportion of infants incorrectly diagnosed as HIV-infected and initiated on ART. While projected cost differences are small, confirmatory testing is cost-saving under a wide range of scenarios in South Africa. Confirmatory testing with ART initiation at the first positive result, as recommended by WHO for EID, should be implemented in settings using NAAT for EID.

## Supporting information

S1 Checklist Consolidated Health Economic Evaluation Reporting Standards (CHEERS) report. (DOCX)

S1 Table ISPOR–SMDM good research practices task force guidelines. Implementation of the International Society for Pharmacoeconomics and Outcomes Research (ISPOR) and Society for Medical Decision Making (SMDM) good research practices within our analysis.
(DOCX)

S2 Table Extended input table. Extensive table containing all input parameters used in the CEPAC–Pediatric model analysis of early infant HIV diagnosis testing in South Africa.
(DOCX)

S1 Text Supplementary appendix. This appendix provides further information on methods and additional model results from extended sensitivity and uncertainty analyses.
(DOCX)

## Abbreviations

ART: antiretroviral therapy
CEPAC: Cost-effectiveness of Preventing AIDS Complications
EID: early infant diagnosis
LE: life expectancy
MTCT: mother-to-child transmission
NAAT: nucleic acid amplification test
OI: opportunistic infection
PMTCT: prevention of mother-to-child transmission
POC: point-of-care
PPV: positive predictive value
WHO: World Health Organization
