# Supplementary material for: The value of confirmatory testing in early infant HIV diagnosis programmes in South Africa: A cost-effectiveness analysis
Source: PLoS Med. 2017 Nov 21;14(11):e1002446. doi: 10.1371/journal.pmed.1002446 (PMC5697827; doi:10.1371/journal.pmed.1002446)
Supplement: S1 Table — Implementation of the International Society for Pharmacoeconomics and Outcomes Research (ISPOR) and Society for Medical Decision Making (SMDM) good research practices within our analysis. (DOCX) [file pmed.1002446.s002.docx]

**S2 Appendix Table: Implementation of the International Society for Pharmacoeconomics and Outcomes Research (ISPOR) and Society for Medical Decision Making (SMDM) Good Research Practices**

| **ISPOR-SMDM Good Research Practice** | **CEPAC-Pediatric model implementation** |
| --- | --- |
| VI-1 The systematic examination and responsible reporting of uncertainty are hallmarks of good modeling practice. All modeling studies should include an uncertainty assessment as it pertains to the decision problem being addressed. | - We conduct and report univariate and multivariate sensitivity analyses for all input parameters, as well as key scenario analyses. We provide these results in text and graphic form in the manuscript, with complete information additionally presented in table form in the supplementary appendix (Tables S1-S4). |
| VI-2 The decision-maker’s role should be considered when presenting uncertainty analyses. The analytic perspective description should include an explicit statement regarding what is assumed about the decision-makers’ power to delay or review decisions and to commission or mandate further research.  --In addition the task force offers an explanation as: “If the decision-maker must make a decision now, has no role in commissioning ... further research ... then the role of uncertainty analysis is limited and the decision should be based on expected values. Nevertheless, decision-makers may want to gauge confidence in the “best choice’s” appropriateness by exploring its robustness to changes in model input [sensitivity].” | - Following both ISPOR/SMDM and CHEERS guidelines (references 20 and 21), we clearly describe our target population, the setting, and the analytic perspective of the analysis. CEPAC analyses are conducted from the healthcare system perspective: to inform either providers or program planners needing to decide which strategy to apply to the population of patients in their care. - We assume that a decision must be made now, with limited ability to commission additional research. We examine both uncertainty and the robustness of the policy conclusion to changes in model input (sensitivity). |
| VI-3 Terminology to describe concepts relating to parameter estimation and representation of uncertainty varies within the health-care decision-modelling field and in comparison to related fields. Authors should be aware of this and seek to carefully define their use of terminology to avoid potential confusion. | - We adhere to terminology recommended by the Task Force, including stochastic uncertainty, parameter uncertainty, and structural uncertainty; univariate and multivariate sensitivity analysis; and scenario analysis. - We indicate explicitly when an examination of the relationship between model inputs and model output is meant to evaluate sensitivity of the “best choice” to these variations vs. true underlying uncertainty in model input parameters. |
| VI-4 All decision models have parameters that need to be estimated. Populating models with parameter estimates should conform to evidence-based medicine principles  --For example, analysts should “seek to incorporate all evidence, rather than selectively picking a single source." | - We seek to identify and incorporate all relevant evidence when estimating parameter inputs. We perform extensive literature searches to inform each model input parameter. - Where many data sources are available to inform estimates, we used the mean of available values (for example, mother-to-child HIV transmission rates with and without ART) for base-case values. For estimates of setting-specific uptake of healthcare services and practices (for example, PMTCT coverage, EID test uptake, result-return rate), we use the most recent available published data from South Africa. For estimates of assay sensitivity and specificity, we use systematic reviews conducted by WHO, updated with expert opinion. - We include additional description of these data sources and methods of data input derivation in Table S2. |

**S3 Appendix Table: Implementation of ISPOR-SMDM Good Research Practices, continued**

| **ISPOR-SMDM Good Research Practice** | **CEPAC-Pediatric model implementation** |
| --- | --- |
| VI-5 Whether employing deterministic methods (point estimate and range) or probabilistic (parameterized distribution), the link to the underlying evidence base should be clear.  VI-6 While completely arbitrary analyses, like presentation of the effect on outputs of varying inputs by ±50%, can be used as a measure of sensitivity; such analyses should not be used to represent uncertainty.  --For example, ranges selected should reflect measures of precision in the estimates, such as 95% confidence intervals, as well as measure of covariance between parameters, where relevant.  Authors state --"Some uncertainty analyses do not require formal ascertainment of parameter uncertainty. These include threshold analyses, where the parameter's value needed to change the decision is identified (please see VI-8 below). This is closely linked to "even if" approaches that identify extreme parameter values that still do not change the decision ... Another form not requiring uncertainty estimation is identification of the quantitative relationships between inputs and outputs (e.g., ...a 10% increase in a particular parameter's value leads to a 20% increase in expected effectiveness and a 5% decrease in expected cost)."  VI-7 Use commonly adopted statistical standards for point and interval estimation (e.g., 95% confidence intervals, or distributions based on agreed statistical methods for a given estimation problem). Where departures from these standards are deemed necessary (or no such standard exists), these should be justified. | - When model input parameters are chosen from a single published study, we use the mean value for the base-case, and vary the parameter through the 95% CI to assess uncertainty (i.e., NAAT specificity, linkage to HIV-care for infected infants; Table S2 and Figure 2). - When model input parameters are taken from the mean of many studies (e.g., MTCT rates), we vary them over the full published range (Table S2 and Figure 2); there is no confidence interval available, as the data were not aggregated in a formal meta-analysis. - Where inputs reflect setting-specific uptake of healthcare services (e.g., PMTCT coverage, EID uptake), or where there are few sources of data, per the Task Force guidelines, we derive a range for sensitivity analyses with the primary intention of reflecting the sensitivity of model conclusions to variations in these values, so that program planners can see the impact for their own programs, rather than to reflect uncertainty in the data estimate pertaining to a single setting. - Several parameters are likely to be correlated (e.g., PMTCT coverage and EID uptake; EID uptake and linkage to ART). Because no single study provided an estimate of covariance between such parameters, we do not include a formal measure of covariance, but we create several scenario analyses that allowed these values to vary in clinically and programmatically plausible ways. |
| VI-8 Where there is very little information on a parameter; adopt a conservative approach such that the absence of evidence is reflected in a very broad range of possible estimates. Never exclude parameters from uncertainty analysis on the grounds that there is insufficient information to estimate uncertainty. | - We vary all input parameters over extreme ranges whilst performing threshold analyses. This includes varying values through the original ranges derived from the data (VI 5-7, above). If this does not lead to a change in policy conclusion, we extend the range further, to identify the value needed to change the decision. This “threshold analysis” or “even if” approach often identifies extreme parameter values; where these are not clinically plausible values, we note this in the manuscript. |

| **ISPOR-SMDM Good Research Practice** | **CEPAC-Pediatric model implementation** |
| --- | --- |
| VI-9 Favor continuous distributions that portray uncertainty realistically over the theoretical range of the parameter. Careful consideration should be given to whether convenient-to-fit but implausible distributions (such as the Triangular) should have a role in PSA. | - We vary all key parameters over a continuous range. |
| VI-10 Correlation among parameters should be considered. Jointly estimated parameters, such as those from a regression analysis, will have direct evidence on correlation which should be reflected in the analysis. Independently estimated parameters will have no such evidence, but this should not necessarily lead to an assumption of independence. Possible approaches are to include a correlation coefficient as a parameter where concern exists that an unknown correlation could be important; or to re-parameterize the model so that that the uncertain parameters can be reasonably assumed to be independent. | - As noted above, we are rarely able to derive multiple related parameters from the same data source, and thus are not able to use published or calculated estimates of correlation. We evaluate the impact of simultaneous variation in multiple parameters in two ways: multivariate sensitivity analyses and clinical scenario analyses (Figure 4 and Table 3, main manuscript, Table S6). In these scenario analyses, we allow multiple parameters to vary together in ways that reflect differences in setting, healthcare system, or biologic properties. |
| VI-11 Where uncertainties in structural assumptions were identified in the process of conceptualizing and building a model, those should be tested in uncertainty analysis. Consideration should be given to opportunities to parameterize these uncertainties for ease of testing. Where it is impossible to perform structural uncertainty analysis, it is important to be aware that this uncertainty may be at least as important as parameter uncertainty. | - The CEPAC-Pediatric model has been internally validated against event rates (opportunistic infections (OIs)) from untreated children, then calibrated to survival data from untreated HIV-infected children and from children treated with ART. In the calibration of outcomes for untreated children, the most influential parameters were monthly rate of CD4% decline, risk of mortality in the 30 days following an OI, and risk of mortality >30 days after an OI (Ciaranello et al. *PLoS ONE* 2013). In the calibration of outcomes for treated children, the most influential parameters were the CD4-independent risks of OI and mortality, a parameter that was built to reflect model structural uncertainty (Ciaranello et al. *AIDS* 2015). We then examine the impact of this uncertainty on our model results and policy conclusions (please see V1-14, below). |
| VI-12 It is appropriate to report both deterministic and probabilistic uncertainty analyses within a single evaluation. Tornado diagrams, threshold plots, or simple statements of threshold parameter values, are all appropriate ways of reporting results from deterministic sensitivity analyses. | - To report the results of our deterministic sensitivity analyses, we include a tornado diagram (Figure 2) and threshold analyses (Figure 2 and text). |
| VI-13 When additional assumptions or parameter values are introduced for purposes of uncertainty analyses; these values should be disclosed and justified. | - The values and data sources for all parameter estimates are listed in Table 1 (main manuscript) and Tables S1 and S2; key inputs are also described in the manuscript text. |

**S3 Appendix Table: Implementation of ISPOR-SMDM Good Research Practices, continued**

| **ISPOR-SMDM Good Research Practice** | **CEPAC-Pediatric model implementation** | |
| --- | --- | --- |
| VI-14 When model calibration is used to derive parameters; uncertainty around the calibrated values should be reported and reflected in deterministic or probabilistic sensitivity analyses, or both. | | - We repeat our policy comparison (EID *with* vs. *without confirmatory testing*), using the 25 best-fitting parameter sets from our analyses for untreated children and for treated children, and find that in all analyses, *with confirmatory testing* remains cost-saving compared to *without confirmatory testing* (Figure S3). |
| VI-15 When the purpose of a probabilistic sensitivity analysis is to guide decisions about acquisition of information to reduce uncertainty; results should be presented in terms of expected value of information. | | - N/a |
| VI-16 For economic studies, when a probabilistic SA is performed without an accompanying expected value of information analysis, options for presenting results include cost-effectiveness acceptability curves and distributions of net monetary benefit or net health benefit. When more than two comparators are involved, curves for each comparator should be plotted on the same graph. | | - N/a |

Abbreviations: **ART**: antiretroviral therapy; **CEPAC**: Cost Effectiveness of Preventing AIDS Complications; **CHEERS**: Consolidated Health Economic Evaluation Reporting Standards; ; **EID**: early infant diagnosis; **HIV**: human immunodeficiency virus **ISPOR**: International Society for Pharmacoeconomics and Outcomes Research; **PMTCT**: Prevention of mother-to-child transmission; **SMDM**: Society for Medical Decision Making; **WHO**: World Health Organization
