# Supplementary material for: The value of confirmatory testing in early infant HIV diagnosis programmes in South Africa: A cost-effectiveness analysis
Source: PLoS Med. 2017 Nov 21;14(11):e1002446. doi: 10.1371/journal.pmed.1002446 (PMC5697827; doi:10.1371/journal.pmed.1002446)
Supplement: S2 Table — Extensive table containing all input parameters used in the CEPAC–Pediatric model analysis of early infant HIV diagnosis testing in South Africa. (DOCX) [file pmed.1002446.s003.docx]

**Supplemental Table S1. Data parameters for CEPAC-Pediatric model (includes those shown in Manuscript Table 1)**

| **Cohort characteristics** | **Value (range for sensitivity analyses)** | | | **Sources** | |
| --- | --- | --- | --- | --- | --- |
| Age, months (SD) | 0 (0) | | | Assumption | |
| Percent male | 48.8% | | | [1] | |
| Mothers with maternal CD4 ≤ 350 cells/µL before ART | 36% (30-50) | | | [2] | |
| Breastfeeding (proportion of all mother-infant pairs) ^a^ | 80% (50-100) | | | Assumption | |
| Exclusively breastfeeding for 1^st^ 6 months | 55% | | | [3,4] | |
| Mixed breastfeeding for 1^st^ 6 months | 25% | | | [3,4] | |
| Replacement feeding from birth | 20% | | | [3,4] | |
| Mean breastfeeding duration, months (SD) | 12 (2) (3-18) | | | Assumption | |
| **Mother-to-child transmission parameters** | **Value (%, range examined)** | | | **Sources** | |
|  | *Maternal CD4≤350 cells/µL* | *Maternal CD4>350 cells/µL* | |  | |
| *Intrauterine (IU)/intrapartum (IP)-- one-time risk* | |  | |  | |
| On ART (60% IU; 40 IP) | 1.0 | 1.0 | | [5–8] | |
| Not on ART (60% IU; 40 IP) | 27 | 17 | | [9–14] | |
| *Postpartum (PP)—monthly risks during breastfeeding* | |  | |  | |
| On ART | 0.19 | 0.19 | | [6–8,14–19] | |
| Not on ART |  |  | |  | |
| Exclusive breastfeeding | 0.76 | 0.24 | | [2,9–14,20] | |
| Mixed or complementary feeding | 1.28 | 0.40 | | [2,9–14,20] | |
| Probability maternal status is known in pregnancy | 100 | 100 | | Assumption | |
| Probability mother on ART in pregnancy and breastfeeding | 90 (40-100) | 90 (40-100) | | [21] | |
| Monthly maternal mortality probability | 0.21 | 0.11 | | [21,22] | |
| **Clinical data: untreated, HIV-infected children** | **Value (%)** | |  | | **Sources** |
| CD4% at infection (SD) | 45 (10) | |  | | [23] |
| Monthly rate of CD4%/CD4 decline, by age |  | |  | |  |
| <3 months of age (CD4%, IU/IP infections only) | 4.00 | |  | | [23] |
| 3-59 months of age (CD4%, IU/IP infections only) | 0.5 | |  | | [23] |
| 0-59 months of age (CD4%, PP infections only) | 0.5 | |  | | [23] |

**Supplemental Table S1 (continued)**

| **Clinical data: untreated, HIV-infected children** | **Value (%)** | **Sources** |
| --- | --- | --- |
| Monthly CD4 decline (cells/μl) stratified by HIV RNA (SD)  >30,000 copies/ml  10,001-30,000 copies/ml  3,001-10,000 copies/ml  500-3,000 copies/ml  <500 copies/ml | 6.4 (0.3)  5.4 (0.2)  4.6 (0.2)  3.7 (0.2)  3.0 (0.3) | [1,23,24]  [1,23,24]  [1,23,24]  [1,23,24]  [1,23,24] |
| Monthly risk of clinical events (range by CD4%) |  |  |
| <60 months of age |  |  |
| WHO Stage 3 event (except tuberculosis) | 3.3-11.6 | [1] |
| WHO Stage 4 event (except tuberculosis) | 1.4-6.4 | [1] |
| Tuberculosis (any body site) | 0.5-3.8 | [1] |
| ≥60 months of age |  |  |
| Mild fungal infection | 1.8-3.1 | [25] |
| Visceral bacterial infection | 0.0-0.7 | [25] |
| WHO Stage 3 or 4 visceral disease | 0.0-1.4 | [25] |
| WHO Stage 3 or 4 mucocutaneous disease | 0.0-2.3 | [25] |
| Other WHO Stage 3 or 4 disease | 0.0-0.7 | [25] |
| Other severe disease | 0.2-1.7 | [25] |
| Other mild disease | 2.4 | [25] |
| Tuberculosis (any body site) | 0.0-1.7 | [25] |
| Risk of death within 30 days of clinical event |  |  |
| <60 months of age |  |  |
| After WHO Stage 3 or 4 event | 13.5 | [1] |
| After TB event | 11.1 | [1] |
| ≥60 months of age |  |  |
| Mild fungal infection | 0.5 | [25] |
| Visceral bacterial infection | 2.9 | [25] |
| WHO Stage 3 or 4 visceral disease | 9.2 | [25] |
| WHO Stage 3 or 4 mucocutaneous disease | 2.4 | [25] |
| Other WHO Stage 3 or 4 disease | 20.0 | [25] |
| Other severe disease | 6.7 | [25] |
| Other mild disease | 0.4 | [25] |
| TB (any body site) | 1.8 | [25] |
| Monthly risk of HIV-related death (range by age, CD4%/CD4, and history of prior OI) | 0.1-40.8 | [1,23,25] |

**Supplemental Table S1 (continued)**

| **Clinical data: untreated, HIV-infected children** | **Value (%)** | **Sources** |
| --- | --- | --- |
| Transition CD4% to Absolute CD4 at 5yrs (SD)  0-4%  5-9%  10-14%  15-19%  20-24%  25-29%  30-35%  >35% | 30 (7.5)  75 (18.75)  150 (37.5)  250 (62.5)  375 (93.7)  550 (137.5)  725 (181.25)  900 (225) | [1,23] |
| Monthly risk of infant mortality among HIV-exposed, uninfected infants | | |
| 0-2 months | 1.0 | [26,27] |
| 3-5 months | 0.4 | [26,27] |
| 6-11 months | 0.3 | [26,27] |
| 12-17 months | 0.1 | [26,27] |
| 18-23 months | 0.1 | [26,27] |
| Monthly risk of non-AIDS related mortality (range by age in yearly intervals, sex) | |  |
| <12 months of age | 0.33-0.42 | [28] |
| 12-60 months of age | 0.02-0.03 | [28] |
| 5-13 years of age | 0.00-0.01 | [28] |
| 13-18 years of age | 0.00-0.01 | [28] |
| >18 years of age | 0.00-1.40 | [28] |

**Supplemental Table S1 (continued)**

| **EID cascade parameters** | **Guideline concordant (%)** | | **Range examined (%)** | **Sources** |
| --- | --- | --- | --- | --- |
| Probability of presenting to a testing visit (%) | 100 | | 0-100 |  |
| Probability of being offered and accepting test (%) | 100 | | 0-100 | Scenario-specific |
| Probability of receiving test results (%) | 100 | | 0-100 | assumptions |
| Delay between primary test and result receipt (SD) | 1 month (0 months) | | 0-5m |  |
| Delay between confirmatory test and result receipt (SD) | 0 months (1 month) | | 0-12m |  |
| **Nucleic Acid Amplification Test (NAAT) assay characteristics** |  | |  |  |
| Sensitivity for IU infection (by age) | 100 (all ages) | | 90-100 (all ages) | [29] |
| Sensitivity for IP infection (by age) | Month 1: 0  Later months: 100 | | Month 1: 0  Later months: 90-100 | [29] |
| Sensitivity for PP infection infants (by time since infection) | Month of infection: 0  Later months: 100 | | Month of infection: 0  Later months: 80-100 | [29] |
| Specificity | 99.6 (all ages) | | 90-100 (all ages) |  |
| **ART outcomes** | | **Value (%)** | | **Sources** |
|  | LPV/r/ABC/3TC  (1^st^-line ART) | | EFV/ AZT/3TC  (2^nd^-line ART) |  |
| ART efficacy: HIV RNA <400c/mL at 24 weeks on ART |  | |  |  |
| Ages 0-59 months | 91 | | 75 | [30,31] |
| Ages 60+ months | 75 | | 75 | [32] |
| CD4%/CD4 gain on suppressive ART (1^st^ 6 months, after 6 months) | | |  |  |
| Ages <60 months (CD4%) | 1.9, 0.4 | | 2.2, 0.7 | [30,31] |
| Ages ≥60 months (CD4 cells/µL) | 67.3, 3.4 | | 67.3, 3.4 | [33] |
| Probability of virologic failure after initial suppression |  | |  |  |
| Any regimen, per month | 0.91 | | 0.91 | [30,31] |
| Relative risk reduction for patients on ART |  | |  |  |
| Risk of opportunistic infection (age 0-13) | 85 | | 85 | [23] |
| Risk of opportunistic infection (age 13+) | 32 | | 32 | [34] |
| Mortality risk (age 0-13) | 90 | | 90 | [23] |
| Mortality risk (age 13+, range by CD4) | 55-96 | | 55-96 | [34] |
| Monthly loss to follow-up after ART initiation (applied lifelong) | 0.2 | | 0.2 | [35,36] |

**Supplemental Table S1 (continued)**

| **Costs** | **Value (2013 USD)** | | **Sources** |
| --- | --- | --- | --- |
| Opportunistic infection care (per event; range by type of OI) |  | |  |
| <60 months of age | 1,240-2,175 (x0.5-2.0) | | [37] |
| ≥60 months of age | 260-865 (x0.5-2.0) | | [38,39] |
| Routine care (per month; range by CD4) | 20-165 (x0.5-2.0) | | [25,39] |
| Care in the last month of life | 645 (x0.5-2.0) | | [25,39] |
| Antiretroviral regimen costs (per month, range by age/weight)^b^ | | |  |
| Zidovudine/lamivudine (pediatric or adult tablets) | 3-8 (x0.5-2.0) | | [40,41] |
| Efavirenz (pediatric or adult tablets) | 3-6 (x0.5-2.0) | | [40,41] |
| Lopinavir/ritonavir (liquid: age <3 years) | 12-24 (x0.5-2.0) | | [40,41] |
| Lopinavir/ritonavir (pediatric or adult tablets) | 13-26 (x0.5-2.0) | | [40,41] |
| Abacavir/lamivudine (pediatric or adult tablets) | | 8-21 (x0.5-2.0) |  |
| Cost of **NAAT** assay^c^ | 25 (10-400) | | Assumption |
| Negative **NAAT** result-return | 1.83 | | Assumption (nurse time x salary) [42] |
| Positive **NAAT** result-return | 3.05 | |  |
| Cost of CD4 test monitoring (annual after 12m ART) | 11.15 (x0.5-2.0) | | [39,43] |
| Cost of HIV VL monitoring (annual after 12m ART) | 40 (x0.5-2.0) | | [39,43] |

Abbreviations: **SD:** standard deviation; **IU:** intrauterine; **IP:** intrapartum; **PP:** postpartum; **ART:** anti-retroviral therapy; **EID:** early infant diagnosis; **HIV:** human immunodeficiency virus; **RNA:** ribonucleic acid; **WHO:** World Health Organization; **TB:** tuberculosis; **OI:** opportunistic infection; **AIDS:** acquired immunodeficiency syndrome; **NAAT:** nucleic acid test; **PCR:** polymerase chain reaction; **LPV/r:** lopinavir/ritonavir; **ABC:** abacavir; **3TC:** lamivudine; **EFV:** efavirenz; **NVP:** nevirapine; **AZT:** zidovudine

a. Exclusive breastfeeding applies (first 6 months of life) in 55%; mixed breastfeeding (first 6 months of life) in 25%; replacement feeding from birth in 20%. After 6 months of age, all infants still breastfeeding are assumed to have complimentary feeding (breastmilk and other liquids/solids).

b. Monthly ART drug doses were calculated for children ages 0-13 years old based on the WHO weight-based dosing recommendations. Daily doses were then multiplied by unit drug costs from the 2014 Clinton Health Access Initiative (CHAI) ARV price list to determine monthly ART costs by age and weight. All children were assumed to receive liquid/syrup drug formulations until age 3 years for lopinavir/ritonavir, and until age 6 months for all other medications, for which dispersible tablets are available. After these ages, children were assumed to transition to pediatric or adult tablet formulations based on weight-based dosing recommendations. Fixed dose combinations were assumed to be used where available.

c. **NAAT** costs include assays, reagents, and personnel time for counseling, blood draws, specimen transport and processing, and quality control.

References

1. Ciaranello AL, Lu Z, Ayaya S, Losina E, Musick B, Vreeman R, et al. Incidence of WHO Stage 3 and 4 Events, Tuberculosis, and Mortality in Untreated, HIV-Infected Children Enrolling in Care Before 1 Year of Age: An Iedea (International Epidemiologic Databases To Evaluate AIDS) East Africa Regional Analysis. Pediatr Infect Dis J. 2014;33(6):623–9.

2. Iliff PJ, Piwoz EG, Tavengwa N V, Zunguza CD, Marinda ET, Nathoo KJ, et al. Early exclusive breastfeeding reduces the risk of postnatal HIV-1 transmission and increases HIV-free survival. Aids [Internet]. 2005;19(7):699–708.

3. Lilian RR, Johnson LF, Moolla H, Sherman GG. A mathematical model evaluating the timing of early diagnostic testing in HIV-exposed infants in South Africa. J Acquir Immune Defic Syndr [Internet]. 2014;67(3):341–8.

4. Kuhn L, Kroon M. Breastfeeding and the 2015 South African guidelines for prevention of mother-to-child transmission of HIV. S Afr J HIV Med. 2015;16(1). Available from: http://dx.doi.org/10.4102/sajhivmed.v16i1.377.

5. Kilewo C, Karlsson K, Ngarina M, Massawe A, Lyamuya E, Swai A, et al. Prevention of Mother-to-Child Transmission of HIV-1 Through Breastfeeding by Treating Mothers With Triple Antiretroviral Therapy in Dar es Salaam, Tanzania: The Mitra Plus Study. JAIDS J Acquir Immune Defic Syndr. 2009;52(3):406–16.

6. Shapiro RL, Hughes MD, Ogwu A, Kitch D, Lockman S, Moffat C, et al. Antiretroviral Regimens in Pregnancy and Breast-Feeding in Botswana. N Engl J Med. 2010;362(24):2282–94.

7. Kesho Bora Study Group, de Vincenzi I.. Triple antiretroviral compared with zidovudine and single-dose nevirapine prophylaxis during pregnancy and breastfeeding for prevention of mother-to-child transmission of HIV-1 (Kesho Bora study): A randomised controlled trial. Lancet Infect Dis. 2011;11(3):171–80.

8. Tonwe-Gold B, Ekouevi DK, Viho I, Amani-Bosse C, Toure S, Coffie PA, et al. Antiretroviral treatment and prevention of peripartum and postnatal HIV transmission in West Africa: Evaluation of a two-tiered approach. PLoS Med. 2007;4(8):1362–73.

9. Fawzi W, Msamanga G, Spiegelman D, Renjifo B, Bang H, Kapiga S. Transmission of HIV-1 through breastfeeding among women in Dar es Salaam. J Acquir Immune Defic Syndr. 2002;31(3):331–8.

10. Petra Study Team. Efficacy of three short-course regimens of zidovudine and lamivudine in preventing early and late transmission of HIV-1 from mother to child in Tanzania, South Africa, and Uganda (Petra study): A randomised, double-blind, placebo-controlled trial. Lancet. 2002;359(9313):1178–86.

11. Leroy V, Karon J, Alioum A, Ekpini E, Meda N, Greenberg A. Twenty-four month efficacy of a maternal short-course zidovudine regimen to prevent mother-to-child transmission of HIV-1 in West Africa. AIDS. 2002;16(4):631–41.

12. Chigwedere P, Seage G, Lee T, Essex M. Efficacy of antiretroviral drugs in reducing mother-to-child transmission of HIV in Africa: a meta-analysis of published clinical trials. AIDS Res Hum Retroviruses. 2008;24(6):827–37.

13. Dabis F, Bequet L, Ekouevi DK, Viho I, Rouet F, Horo A, et al. Field efficacy of zidovudine, lamivudine and single-dose nevirapine to prevent peripartum HIV transmission. AIDS. 2005;19(3):309–18.

14. Thior I, Lockman S, Smeaton LM, Shapiro RL, Wester C, Heymann SJ, et al. Breastfeeding Plus Infant Zidovudine Prophylaxis for 6 Months vs Formula Feeding Plus Infant Zidovudine for 1 Month to Reduce Mother-to-Child HIV Transmission in Botswana. Jama. 2006;296(7):794.

15. Peltier CA, Ndayisaba GF, Lepage P, van Griensven J, Leroy V, Pharm CO, et al. Breastfeeding with maternal antiretroviral therapy or formula feeding to prevent HIV postnatal mother-to-child transmission in Rwanda. AIDS. 2009;23(April):2415–23.

16. Palombi L, Marazzi MC, Voetberg A, Magid NA. Treatment acceleration program and the experience of the DREAM program in prevention of mother-to-child transmission of HIV. AIDS. 2007;21(Suppl 4):S65–71.

17. Chasela C, Hudgens M, Jamieson D, Kayira D, Hosseinipour M, Kourtis A. Maternal or infant antiretroviral drugs to reduce HIV-1 transmission. N Engl J Med. 2010;362(24):2271–81.

18. Vyankandondera J, Luchters S, Hassink E. Reducing risk of HIV-1 transmission from mother to infant through breastfeeding using antiretroviral prophylaxis in infants (SIMBA-study, Abstract N°LB7). 2nd International AIDS Society Conference on HIV pathogenesis, treatment and prevention; Paris, France 2003. Available at: http://www.iasociety.org/Default.aspx?pageId=11&abstractId=11061. Accessed on 7 July 2017..

19. Thomas T, Masaba R, Borkowf C, Ndivo R, Zeh C, Misore A. Triple-antiretroviral prophylaxis to prevent mother-to-child HIV transmission through breastfeeding--the Kisumu Breastfeeding Study, Kenya: a clinical trial. PLoS Med. 2011;8(3):e1001015.

20. Kuhn L, Aldrovandi GM, Sinkala M, Kankasa C, Semrau K, Mwiya M, et al. Effects of Early, Abrupt Weaning on HIV-free Survival of Children in Zambia. N Engl J Med. 2008;359(2):130–41.

21. World Health Organization. Progress report on the global plan towards the elimination of new HIV infections among children and keeping their mothers alive [Internet]. Geneva, Switzerland; 2015. Available from: http://www.unaids.org/sites/default/files/media_asset/JC2774_2015ProgressReport_GlobalPlan_en.pdf.

22. Ciaranello A, Myer L, Kelly K, Christensen S, Daskilewicz K, Doherty K. Point-of-Care CD4 Testing to Inform Selection of Antiretroviral Medications in South African Antenatal Clinics: A Cost-Effectiveness Analysis PLoS One. 2015; 10(3): e0117751. https://doi.org/10.1371/journal.pone.0117751

23. Ciaranello AL, Morris BL, Walensky RP, Weinstein MC, Ayaya S, Doherty K, et al. Validation and calibration of a computer simulation model of pediatric HIV infection. PLoS One. 2013;8(12):1–13.

24. Mellors J, Munoz A, Giorgi J, Margolick J, Tassoni C. Plasma viral load and CD4+ lymphocytes as prognostic markers of HIV-1 infection. Ann Intern Med. 1997;126:946–54.

25. Holmes C, Wood R, Badri M. CD4 decline and incidence of opportunistic infections in Cape Town, South Africa: Implications for prophylaxis and treatment. J Acquir Immune Defic Syndr. 2006;42:464–9.

26. Marston M, Becquet R, Zaba B, Moulton LH, Gray G, Coovadia H, et al. Net survival of perinatally and postnatally HIV-infected children: A pooled analysis of individual data from sub-Saharan Africa. Int J Epidemiol. 2011;40(2):385–96.

27. Becquet R, Marston M, Dabis F, Moulton LH, Gray G, Coovadia HM, et al. Children who acquire hiv infection perinatally are at higher risk of early death than those acquiring infection through breastmilk: A meta-analysis. PLoS One. 2012;7(2).

28. United Nations. World Population Prospects: The 2008 Revision New York2009 [cited 2017 7 July]. Available from: http://www.un.org/esa/population/publications/wpp2008/wpp2008_highlights.pdf.

29. Mallampati D, Ford N, Hanaford A, Sugandhi N, Penazzato M. Performance of virological testing for early infant diagnosis: A systematic review. J Acquir Immune Defic Syndr. 2017;160(2012):1.

30. Violari A, Paed FC, Lindsey JC, Sc D, Hughes MD, Ph D, et al. Nevirapine versus Ritonavir-Boosted Lopinavir for HIV-Infected Children. N Engl J Med. 2012;366(25):2380–9.

31. Palumbo P, Lindsey J, Hughes M. Antiretroviral treatment for children with peripartum nevirapine exposure. N Engl J Med. 2010;363:1510–20.

32. Babiker A, Castro nee Green H, Compagnucci A, Fiscus S, Giaquinto C, Gibb D. First-line antiretroviral therapy with a protease inhibitor versus non-nucleoside reverse transcriptase inhibitor and switch at higher versus low viral load in HIV-infected children: an open-label, randomised phase 2/3 trial. Lancet Infect Dis. 2011;11(4):273–83.

33. Tuboi S, Brinkhof M, Egger M, Stone R, Braistein P, Nash D. Discordant responses to potent antiretroviral treatment in previously naive HIV-1 infected adults initiating treatment in resource-constrained countries: the antiretroviral threapy in low income counties collaboration. J Acquir Immune Defic Syndr. 2007;45(1):52–9.

34. Losina E, Yazdanpanah Y, Deuffic-Burban S, Wang B, Wolf LL, Messou E, et al. The independent effect of highly active antiretroviral therapy on severe opportunistic disease incidence and mortality in HIV-infected adults in Cote d’Ivoire. Antivir Ther. 2007;12(4):543–51.

35. Ciaranello AL, Chang Y, Margulis A V, Bassett I V, Losina E, Rochelle P. Effectiveness of Pediatric Antiretroviral Therapy in Resource-limited Settings: A Systematic Review and Meta-analysis. Clin Infect Dis. 2009;49(12):1915–27.

36. Rosen S, Fox MP. Retention in HIV care between testing and treatment in sub-Saharan Africa: a systematic review. PLoS Med. 2011 Jul;8(7):e1001056.

37. Thomas L. Costing of HIV/AIDS services at a tertiary level hospital in Gauteng Province. University of Witwatersrand, South Africa; 2006.

38. Goldie SJ, Yazdanpanah Y, Losina E, Weinstein MC, Anglaret X, Walensky RP, et al. Cost-Effectiveness of HIV Treatment in Resource-Poor Settings — The Case of Côte d’Ivoire. N Engl J Med. 2006;355(11):1141–53.

39. Cleary S, Okorafor OA, Chitha W, Boulle A, Jikwana S. Financing antiretroviral treatment and primary health care services. South African Heal Rev. 2005;58–74.

40. Clinton Health Access Initiative. 2016 Antiretroviral (ARV) CHAI reference price list. 2016;(November):2015–6. Available from: http://www.clintonhealthaccess.org/content/uploads/2016/11/2016-CHAI-ARV-Reference-Price-List_FINAL.pdf (Accessed March 03, 2017)

41. Doherty K, Essajee S, Penazzato M, Holmes C, Resch S, Ciaranello A. Estimating age-based antiretroviral therapy costs for HIV-infected children in resource-limited settings based on World Health Organization weight-based dosing recommendations. BMC Health Serv Res. 2014;14(1):201.

42. Bassett I V, Giddy J, Nkera J, Wang B, Losina E, Lu Z, et al. Routine voluntary HIV testing in Durban, South Africa: the experience from an outpatient department. J Acquir Immune Defic Syndr. 2007;46(2):181–6.

43. Group NHTW. National Health Laboratory Service List of Diagnostic Tests. 2012.
