# Supplementary material for: The value of confirmatory testing in early infant HIV diagnosis programmes in South Africa: A cost-effectiveness analysis
Source: PLoS Med. 2017 Nov 21;14(11):e1002446. doi: 10.1371/journal.pmed.1002446 (PMC5697827; doi:10.1371/journal.pmed.1002446)
Supplement: S1 Text — This appendix provides further information on methods and additional model results from extended sensitivity and uncertainty analyses. (DOCX) [file pmed.1002446.s004.docx]

**The Value of Confirmatory Testing in Early Infant HIV Diagnosis (EID) Programmes Supplementary Appendix**

Lorna Dunning, MBiochem, MPH

Jordan A. Francke, BA
Divya Mallampati, MD, MPH
Rachel L. MacLean, BA
Martina Penazzato, MD, PhD
Taige Hou, BSc
Landon Myer, MBChB, PhD
Elaine J. Abrams, MD
Rochelle P. Walensky, MD, MPH
Valériane Leroy, MD, PhD
Kenneth A. Freedberg, MD, MSc
Andrea Ciaranello, MD, MPH

This appendix offers supplementary digital content in addition to the methodology described in the manuscript, and provides additional model results from extended sensitivity and uncertainty analyses.

**METHODS**

**Model structure**

The structure of the CEPAC-Pediatric model has been reported in multiple manuscripts, reflecting the modeling of HIV disease progression in the absence of ART and for infants receiving treatment (1–3). Full details of model structure, examples of source code along with data sources and a proposals for collaborations are available on the CEPAC website <http://www.massgeneral.org/mpec> and web2.research.partners.org/cepac In this appendix, we discuss key features of the CEPAC-Pediatric model relevant to early infant HIV diagnosis (EID) and describe our approach to addressing uncertainty in model based analyses.

*Maternal cohort characteristics and mother-to-child HIV transmission (MTCT)*

The CEPAC-Pediatric model is a computer-based, Monte Carlo simulation model of the progression and outcomes of HIV disease in a hypothetical cohort of infants. Infants enter the model at birth and are simulated until death; they undergo monthly transitions from one health state to another reflecting the natural history of illness and the impact of antiretroviral therapy (ART) on disease progression. A set of estimated probabilities assigned by a random number generator are used to determine the sequence of movements between health states for each individual patient. Reflecting World Health Organization (WHO) guidelines for EID, we simulated only HIV-exposed infants (those born to women with HIV infection identified during pregnancy) (4). At birth, infants are assigned key maternal characteristics, including knowledge of HIV status (reflecting HIV testing in pregnancy), receipt of ART during pregnancy and breastfeeding (prevention of mother-to-child transmission [PMTCT] coverage), and maternal CD4 count. Maternal CD4, a proxy for disease stage, and availability of ART during pregnancy determine the risk of HIV transmission during three time periods: intrauterine (one-time risk), intrapartum (one-time risk), and postpartum (monthly risk until weaning).

*Untreated pediatric HIV infection*
At the time of HIV infection, infants are randomly assigned to a health state drawn from distributions of HIV RNA and CD4 levels; the model uses CD4 percentage (CD4%) for children <5 years old and absolute CD4 count thereafter (5). Without effective ART, CD4% or CD4 count declines monthly. In each month, current age and CD4%/CD4 determine risks of opportunistic infections and death (Table S1 and Supplementary Information S4). The model tracks true CD4%/CD4 and HIV RNA level, although clinical decisions are made based on observed information, such as symptomatic illness, CD4%/CD4 count, or RNA levels. These criteria are measured according to user-specified laboratory monitoring strategies.

*Diagnosis of pediatric HIV infection*

We simulated EID programs with and without confirmatory testing. In the base case, all EID testing occurred at 6 weeks of age, per current WHO guidelines (5). In later scenario analyses, we included EID testing at birth and six weeks, as conditionally recommended by the WHO for better-resourced programs and implemented in South Africa (6). In all strategies, previously undiagnosed HIV-infected children who developed an opportunistic infection at any age also presented to care and underwent diagnostic testing (NAAT if <12 months of age, HIV antibody if ≥12 months of age). The CEPAC-paediatric model structure captures competing clinical risks and their associated healthcare costs. Uninfected infants remain at risk of postnatal HIV infection through breastfeeding whilst HIV-infected infants may acquire OIs allowing for HIV diagnosis, but increasing the morbidity of affected infants. HIV-infected infants are at greatest risk of mortality before EID testing and between testing and ART initiation.

*False-positive test results*

We simulate ART initiation after the first positive EID assay result is received, with ART cessation if a confirmatory assay is subsequently negative. A third assay is used for confirmation of negative status as recommended by WHO guidelines. HIV-infected infants therefore do not delay ART initiation for a confirmatory test result. Infants who receive a false positive initial NAAT result and become infected in the waiting period between result return and retesting receive a true positive confirmatory test result (if confirmatory testing occurred at least 1 month from the time of infection) and therefore remain on treatment. We assumed that infants initiating care after a false positive result would accrue HIV care costs, laboratory monitoring costs, and ART costs (but not costs related to opportunistic infections) for 10 years in the base case (varied in sensitivity analyses). Although we included the costs of ART toxicity for HIV-uninfected infants on ART, we excluded clinical impacts for these patients, such as morbidity or mortality related to ART toxicity and stigma or reduced quality of life.

*NAAT sensitivity and specificity*

We modeled NAAT sensitivity as a function of time since infection to reflect the threshold of viremia necessary for detection. NAAT at birth detected intrauterine but not intrapartum infection, and NAAT at 6 or 10 weeks detected intrauterine infection, intrapartum infection, and postpartum infections occurring at least 2 weeks prior (Manuscript Table 1). Specificity, the probability that the NAAT assay will correctly identify a truly uninfected infant as uninfected, is able to be specified by age and HIV test in the model. We assumed conditional independence of the primary and confirmatory NAATs in the base case as WHO guidelines strongly recommend a second specimen is taken for the confirmatory test (4). We varied this assumption in sensitivity analyses.

*EID uptake, result return, and linkage to care*

The model permits users to specify the probabilities that infants are brought by caregivers to each EID testing visit, that test results are returned, and that infants receiving a positive test result will initiate ART. We set these probabilities to 100% in the base case to examine the full potential impact of each testing strategy, and varied them widely in sensitivity analyses.

*Treated pediatric HIV infection*As per WHO guidelines, all infected patients initiate first-line ART upon detection of infection and linkage to HIV care. Each ART regimen has a specified efficacy which defines the probability of suppressing HIV RNA to <400 copies/mL by 24 weeks. Following this initial suppression, there is a monthly risk of late virologic failure. Each regimen also confers monthly medication costs, as well as gains in CD4% or CD4 count for children with suppressed HIV RNA. In each month, children can remain in care or become lost to follow-up (with potential for returning to care following a severe OI); if children are lost to follow-up, they are assumed to stop ART and experience virologic failure. Once virologic failure occurs, HIV RNA slowly rises to a “set point” that is determined as a function of HIV RNA level after initial infection; after a user-specified time period, CD4%/CD4 also begins to decline at the same rate as for patients not receiving ART. For all patients on ART, regardless of HIV RNA suppression, the model includes a CD4-independent reduction in the risk of mortality and opportunistic infection for children on ART, as observed in adults (2,7).

Although the model is able to simulate up to ten lines of ART administered sequentially, only two are used in this analysis, reflecting current treatment guidelines in South Africa and many other resource-limited settings. Infants are evaluated at each clinic visit for switching or stopping their ART regimen. We assign clinical criteria (number and type of opportunistic infections), immunologic criteria (decline in CD4% or CD4 count), or virologic criteria (increase in HIV RNA) by which ART failure is detected, as well as the type and frequency of monitoring and confirmatory testing. After observed failure, patients can be switched to the next available line of therapy.

**Model outcomes**We calculate incremental cost-effectiveness ratios (ICERs) as change in cost divided by change in life expectancy. This approach captures the impact of longer survival on both clinical outcomes and costs. For each simulated infant, the model tracks clinical events (HIV infection at birth or through breastfeeding, mortality, OIs among infected patients) and the amount of time spent in each health state. Patients accrue a monthly routine care cost, based on current CD4 and age. Large cohorts (30 million-300 million total patients, comprised of both HIV-infected and HIV-uninfected patients) are simulated in order to generate stable model outcomes. Once the entire cohort has been simulated, summary statistics are tallied, including number and type of clinical events, the proportion alive each month, health care costs in each month, and life expectancy (mean for the entire birth cohort). For EID analyses, the model also reports the number of HIV-infected infants detected and not detected at key time points, the number of each type of EID assay performed, false positive and false negative EID results, and EID assay costs.

*Life Expectancy*The model records true infection status for all infants. Life expectancy in years is projected separately for HIV-infected infants and for the complete birth cohort of HIV-exposed infants, which includes both HIV-infected and HIV-uninfected infants. We present only the LE for HIV-infected infants and that of the whole cohort in the main manuscript. There are several other key populations of interest. LE for HIV-uninfected children is based on monthly mortality risks from South African life tables, excluding mortality from HIV and war, from the United Nations World Population Prospects Report. When limited to infants who never become infected, the model projects an average LE of 64.8 years. Previous CEPAC-Pediatric analyses have also evaluated the LE of HIV-infected infants who survive to remain in care and are virologically suppressed on LPV/r at 12 months of age (8). Projecting forward, LE for these infants was found to be between 36-40 years dependent upon the ART regime followed. The LE presented in the main manuscript for the entire cohort of HIV-infected infants is substantially decreased because of infants who die before ART initiation.

**Model input data (Appendix Tables S1 and S2)**

For HIV-exposed, uninfected children, we used age-stratified mortality rates from pooled UNAIDS data. Risks of MTCT were from published trials among breastfeeding women and infants in sub-Saharan Africa (9–15). NAAT assay sensitivity, specificity, and costs were from WHO systematic reviews, published data, and expert opinion (16). For untreated HIV-infected children, we derived rates of CD4% or CD4 decline, opportunistic infections, and death through age 13 years from the International Epidemiologic Database to Evaluate AIDS (IeDEA) East African region, and at ages ≥13 from the Cape Town AIDS Cohort (17,18). ART efficacy data were from the P1060 trial and PENPACT-1 trials (9,10).

**Model calibration**

*Natural history model calibration*

The model has been internally validated against input data from a cohort of HIV-infected children included in the IeDEA East Africa region who were not treated with ART, then calibrated against survival data from perinatally infected infants from the UNAIDS Child Survival Group [1,6,13,16]

*On-ART model calibration*

On-ART model calibration has been described in previous work, but is clarified again here, as it has been shown to have large implications in cost effectiveness of ART regimens (1,2). Adults on ART may have reduced risks of OIs and death, regardless of HIV RNA suppression and independent of current CD4 count (7). There are no data on a similar effect in children under the age of 13. We used an "ART-associated CD4-independent reduction in OI and mortality risk" in children in order to calibrate the model to observed OI and mortality rates from the P1060 trial (2,10). We first attempted to match the mortality rates observed in the P1060 trial (3.29/100PY). The best fit to the P1060-observed mortality rate was found with relative reductions of 85-95% in the relative risk of mortality that occurs outside of the 30 days immediately following an opportunistic infection. Holding the relative risk reduction in mortality at 90% (the midpoint of this range), we next compared model-generated OI rates using these multipliers to P1060-observed rates of WHO stage 3, WHO stage 4, and tuberculosis events. These were found to match most closely when the relative reduction in opportunistic infection risk was 85%. Finally, we compared the projected life expectancies to results from relative reductions in OI risk of 85-95% and relative reductions in mortality risk of 90-95% (17). There are no empiric data to inform the life expectancy of HIV-infected African children treated with modern ART regimens. Based on projected results for adults and in consultation with clinical experts in pediatric HIV care, we felt that life expectancies in the 27-28-year range for children surviving to 12 months of age before ART, as observed with relative risk reductions of 85% and 90%, were most reasonable. We thus selected relative risk reductions of 90% for mortality and 85% for opportunistic infection as our final calibrated parameters. In previous analyses, variation in this parameter has led to wide variations in projected life expectancy and lifetime costs. In a previous analysis of test timing and frequency for EID testing we found that if the ART-associated CD4-independent reduction was smaller, EID testing became less cost-effective compared to no EID program (19).

To summarize, we have previously conducted three types of calibration and validation: First, we calibrated model-projected survival for untreated HIV-infected infants to published data from 12 PMTCT follow-up studies in 8 sub-Saharan Africa countries. This exercise allowed us to adjust key parameters to produce more generalizable results and to use a formal likelihood-based approach to quantify the degree to which model projections match observed data (also in Ciaranello *et al., PLoS ONE*, 2013). Second, we calibrated model-projected OI and mortality rates among HIV-infected infants treated with ART to published data from the P1060 trial (Ciaranello *et al., AIDS,* 2015, with key details in the Appendix; available freely at https://www.ncbi.nlm.nih.gov/pmc/articles/PMC4536981/). Third, we did an internal validation of model-projected OIs and survival for untreated HIV-infected infants. We compared model projections to the data from the IeDEA research consortium that were used for model inputs. This ensures that the mathematical structure of the model is accurate (Ciaranello *et al., PLoS ONE*, 2013; open access).

**Sensitivity analyses**

The main manuscript contains selected univariate and multivariate sensitivity analyses, identifying the thresholds for key parameters at which clinical or cost-effectiveness results change (20,21). This appendix contains additional analyses not shown in the main manuscript.

Uncertainty analyses
The univariate and multivariate sensitivity analyses described above reveal the sensitivity of policy conclusions to variations in key parameters through a wide range of plausible values. Evaluation of the uncertainty in the primary data estimates requires separate approaches. To evaluate parameter uncertainty, we varied key parameters through reported 95% confidence intervals (or range or interquartile ranges, if 95% confidence intervals were not available; Table 1 (main manuscript), S4; extended input table and Tables S1 and S2 Appendix). For most parameters, this interval fell well within the range examined in sensitivity analyses.

To evaluate both model structural uncertainty and parameter uncertainty, we expanded upon previously conducted formal model validation and calibration analyses (1,2,17). For untreated HIV-infected infants, this previous work identified 11 uncertain but influential parameters: CD4% at birth, monthly rate of CD4% decline from 0-2 months and from 2-60 months of age, risk of OI, risk of death </=30 days after OI, and risk of mortality >30 days after OI for each of six age groups. OI and mortality risks were parameterized as multipliers, applied to primary data derived from the IeDEA cohort. For treated children, the most influential and uncertain parameters were the CD4-independent, ART-associated risk of OI and mortality >30 days after OI.

We repeated the base-case policy analyses comparing *with* and *without confirmatory testing* using the five best-fitting parameter sets for the 11 parameters influencing untreated survival (holding treated parameters equal to the base case), the five best-fitting parameter sets for the two parameters influencing treated survival (holding untreated parameters equal to the base case value), and all 25 combinations of these parameters sets. Because the life expectancy for with and without confirmatory testing was equivalent (holding the base-case assumption that ART was initiated after the first positive test result, and excluding any harms from incorrect ART initiation in uninfected children), we display only the cost outcomes for these uncertainty analyses. Figure S3 shows a graph of the difference in cost between the with and without confirmatory testing strategies for each parameter set. In all of these analyses, with confirmatory testing remained cost-saving compared to without confirmatory testing.

**Supplemental Table 1. Data parameters for CEPAC-Pediatric model (includes those shown in Manuscript Table 1)**

| **Cohort characteristics** | **Value (range for sensitivity analyses)** | | **Sources** |  |
| --- | --- | --- | --- | --- |
| Age, months (SD) | 0 (0) | | Assumption |  |
| Percent male | 48.8% | | (17) |  |
| Mothers with maternal CD4 ≤ 350 cells/µL before ART | 36% (30-50) | | (22) |  |
| Breastfeeding (proportion of all mother-infant pairs) ^a^ | 80% (50-100) | | Assumption |  |
| Exclusively breastfeeding for 1^st^ 6 months | 55% | | (23,24) |  |
| Mixed breastfeeding for 1^st^ 6 months | 25% | | (23,24) |  |
| Replacement feeding from birth | 20% | | (23,24) |  |
| Mean breastfeeding duration, months (SD) | 12 (2) (3-18) | | Assumption |  |
| **Mother-to-child transmission parameters** | **Value (%, range examined)** | | **Sources** |  |
|  | *Maternal CD4≤350 cells/µL* | *Maternal CD4>350 cells/µL* |  |  |
| *Intrauterine (IU)/intrapartum (IP)-- one-time risk* | |  |  |  |
| On ART (60% IU; 40 IP) | 1.0 | 1.0 | (11,14,25,26) |  |
| Not on ART (60% IU; 40% IP) | 27 | 17 | (12,13,15,27–29) |  |
| *Postpartum (PP)—monthly risks during breastfeeding* | |  |  |  |
| On ART | 0.19 | 0.19 | (14,25,26,29–34) |  |
| Not on ART |  |  |  |  |
| Exclusive breastfeeding | 0.76 | 0.24 | (12,13,15,22,27–29,35) |  |
| Mixed or complementary feeding | 1.28 | 0.40 | (12,13,15,22,27–29,35) |  |
| Probability maternal status is known in pregnancy | 100 | 100 | Assumption | |
| Probability mother on ART in pregnancy and breastfeeding | 90 (40-100) | 90 (40-100) | (36) | |
| Monthly maternal mortality probability | 0.21 | 0.11 | (36,37) | |
| **EID cascade parameters** | **Guideline concordant** | **Range examined** | **Sources** |  |
| Probability of presenting to a testing visit (%) | 100 | 0-100 |  |  |
| Probability of being offered and accepting test (%) | 100 | 0-100 | Scenario-specific |  |
| Probability of receiving test results (%) | 100 | 0-100 | assumptions |  |
| Delay between primary test and result receipt (SD) | 1 month (0 months) | 0-5m |  |  |
| Delay between confirmatory test and result receipt (SD) | 0 months (1 month) | 0-12m |  |  |
| Probability of linking to care/ART after diagnosis (%) | 100 | 0-100 |  |  |

**Supplemental Table 1 (continued)**

| **Clinical data: untreated, HIV-infected children** | **Value (%)** | **Sources** |
| --- | --- | --- |
| CD4% at infection (SD) | 45 (10) | (2) |
| Monthly risk of clinical events (range by CD4%) |  |  |
| <60 months of age |  |  |
| WHO Stage 3 event (except tuberculosis) | 3.3-11.6 | (17) |
| WHO Stage 4 event (except tuberculosis) | 1.4-6.4 | (17) |
| Tuberculosis (any body site) | 0.5-3.8 | (17) |
| ≥60 months of age |  |  |
| Mild fungal infection | 1.8-3.1 | (18) |
| Visceral bacterial infection | 0.0-0.7 | (18) |
| WHO Stage 3 or 4 visceral disease | 0.0-1.4 | (18) |
| WHO Stage 3 or 4 mucocutaneous disease | 0.0-2.3 | (18) |
| Other WHO Stage 3 or 4 disease | 0.0-0.7 | (18) |
| Other severe disease | 0.2-1.7 | (18) |
| Other mild disease | 2.4 | (18) |
| Tuberculosis (any body site) | 0.0-1.7 | (18) |
| Risk of death within 30 days of clinical event |  |  |
| <60 months of age |  |  |
| After WHO Stage 3 or 4 event | 13.5 | (17) |
| After TB event | 11.1 | (17) |
| ≥60 months of age |  |  |
| Mild fungal infection | 0.5 | (18) |
| Visceral bacterial infection | 2.9 | (18) |
| WHO Stage 3 or 4 visceral disease | 9.2 | (18) |
| WHO Stage 3 or 4 mucocutaneous disease | 2.4 | (18) |
| Other WHO Stage 3 or 4 disease | 20.0 | (18) |
| Other severe disease | 6.7 | (18) |
| Other mild disease | 0.4 | (18) |
| TB (any body site) | 1.8 | (18) |
| Monthly risk of HIV-related death (range by age, | 0.1-40.8 | (2,17,18) |
| CD4%/CD4, and history of prior OI) |  |  |

**Supplemental Table 1 (continued)**

| **Clinical data: untreated, HIV-infected children** | **Value (%)** | | | **Sources** |
| --- | --- | --- | --- | --- |
| Monthly risk of infant mortality among breastfed, HIV-exposed, uninfected infants | | | | |
| 0-2 months | 1.0 | | | (38,39) |
| 3-5 months | 0.4 | | | (38,39) |
| 6-11 months | 0.3 | | | (38,39) |
| 12-17 months | 0.1 | | | (38,39) |
| 18-23 months | 0.1 | | | (38,39) |
| **Monthly risk of non-AIDS related mortality (range by age in yearly intervals, sex)** | | | |  |
| <12 months of age | 0.33-0.42 | | | (40) |
| 12-60 months of age | 0.02-0.03 | | | (40) |
| 5-13 years of age | 0.00-0.01 | | | (40) |
| 13-18 years of age | 0.00-0.01 | | | (40) |
| >18 years of age | 0.00-1.40 | | | (40) |
| **Nucleic Acid Amplification Test (NAAT) assay characteristics** | **Base case value (%)** | | **Range examined (%)** | **Sources** |
| Sensitivity for IU infection (by age) | 100 (all ages) | | 90-100 (all ages) | (41) |
| Sensitivity for IP infection (by age) | Month 1: 0  Later months: 100 | | Month 1: 0  Later months: 90-100 | (41) |
| Sensitivity for PP infection infants (by time since infection) | Month of infection: 0  Later months: 100 | | Month of infection: 0  Later months: 80-100 | (41) |
| Specificity | 99.6 (all ages) | | 90-100 (all ages) |  |
| **ART outcomes** | | **Value (%)** | | **Sources** |
|  | LPV/r/ABC/3TC  (1^st^-line ART) | | EFV/ AZT/3TC  (2^nd^-line ART) |  |
| ART efficacy: HIV RNA <400c/mL at 24 weeks on ART |  | |  |  |
| Ages 0-59 months | 91 | | 75 | (9,10) |
| Ages 60+ months | 75 | | 75 | (42) |
| CD4%/CD4 gain on suppressive ART (1^st^ 6 months, after 6 months) | | |  |  |
| Ages <60 months (CD4%) | 1.9, 0.4 | | 2.2, 0.7 | (9,10) |
| Ages ≥60 months (CD4 cells/µL) | 67.3, 3.4 | | 67.3, 3.4 | (43) |
| Probability of virologic failure after initial suppression |  | |  |  |
| Any regimen, per month | 0.91 | | 0.91 | (9,10) |

**Supplemental Table 1 (continued)**

| **ART outcomes (continued)** | **Value (%)** | **Sources** |  |
| --- | --- | --- | --- |
| Relative risk reduction for patients on ART |  |  | |
| Risk of opportunistic infection (age 0-13) | 85 | (1) |  |
| Risk of opportunistic infection (age 13+) | 32 | (7) |  |
| Mortality risk (age 0-13) | 90 | (1) |  |
| Mortality risk (age 13+, range by CD4) | 55-96 | (7) |  |
| Monthly loss to follow-up after ART initiation (applied lifelong) | 0.2 | (44,45) |  |
| **Costs** | **Value (2013 USD)** | **Sources** |  |
| Opportunistic infection care (per event; range by type of OI) |  |  |  |
| <60 months of age | 1,240-2,175 (x0.5-2.0) | (46) |  |
| ≥60 months of age | 260-865 (x0.5-2.0) | (47,48) |  |
| Routine care (per month; range by CD4) | 20-165 (x0.5-2.0) | (18,48) |  |
| Care in the last month of life | 645 (x0.5-2.0) | (18,48) |  |
| Antiretroviral regimen costs (per month, range by age/weight)^b^ | 3-26 (x0.5-2.0) | (49,50) |  |
| Cost of **NAAT** assay^c^ | 25 (10-400) | Assumption |  |
| Negative **NAAT** result-return | 1.83 | Assumption (nurse time x salary) (51) |  |
| Positive **NAAT** result-return | 3.05 |  |  |

Abbreviations: **SD:** standard deviation; **IU:** intrauterine; **IP:** intrapartum; **PP:** postpartum; **ART:** anti-retroviral therapy; **EID:** early infant diagnosis; **HIV:** human immunodeficiency virus; **RNA:** ribonucleic acid; **WHO:** World Health Organization; **TB:** tuberculosis; **OI:** opportunistic infection; **AIDS:** acquired immunodeficiency syndrome; **NAAT:** nucleic acid test; **PCR:** polymerase chain reaction

a. Exclusive breastfeeding applies (first 6 months of life) in 55%; mixed breastfeeding (first 6 months of life) in 25%; replacement feeding from birth in 20%. After 6 months of age, all infants still breastfeeding are assumed to have complimentary feeding (breastmilk and other liquids/solids).

b. Monthly ART drug doses were calculated for children ages 0-13 years old based on the WHO weight-based dosing recommendations. Daily doses were then multiplied by unit drug costs from the 2014 Clinton Health Access Initiative (CHAI) ARV price list to determine monthly ART costs by age and weight. All children were assumed to receive liquid/syrup drug formulations until age 3 years for lopinavir/ritonavir, and until age 6 months for all other medications, for which dispersible tablets are available. After these ages, children were assumed to transition to pediatric or adult tablet formulations based on weight-based dosing recommendations. Fixed dose combinations were assumed to be used where available.

c. **NAAT** costs include assays, reagents, and personnel time for counseling, blood draws, specimen transport and processing, and quality control.

**Supplemental Table 2: Sources of data and ranges for sensitivity analyses and uncertainty analyses**

| **CEPAC Input parameters** | | | |
| --- | --- | --- | --- |
| **Point Estimate** | **Source** | **Uncertainty Analysis Range** | **Sensitivity Analysis**  **Range** |
| **Cohort Characteristics** | | | |
| **Gender** | | | |
| 48.8% | Ciaranello et al. 2014 PIDJ – Cohort of HIV-infected infants from IeDEA east Africa  Le Roux et al 2015 Lancet Global Health – Cohort of South African infants recruited to the Drakenstein Child health Study: 53.4% | 48.8-53.4% | N/A |
| **% women with CD4<350** | | | |
| 36% | Point estimate taken from *Iliff AIDS 2005*  Worst case published 2015 by Myer et al. HIV Medicine: 49%  Moodley et al. BMC Pregnancy Childbirth: 42% | 29.9-50% | 30-50% |
| **Breastfeeding duration** | | | |
| 12 (2) | “National consolidated guidelines for the prevention of mother-to-child transmission of HIV (PMTCT) and the management of HIV in children, adolescents and adults” Department of Health: South Africa 2014 suggest HIV-exposed infants should be exclusively breast fed for at least 6 months, recommended breastfeeding timeline for 12 months and can continue for up to 2 years. | 6-24m | 3-24m |
| **PMTCT/EID Cascade Parameters** | | | |
| **Probability maternal status is known in pregnancy** | | | |
| 100% | Modeled population – EID offered to infants with known HIV-exposure | n/a |  |
| **Probability mother is receiving ART during pregnancy and breastfeeding** | | | |
| 90% | WHO “Progress report on the global plan towards the elimination of new HIV infections among children and keeping their mothers alive”2015 estimate for maternal ART coverage in South Africa  Best case- Moodley et al. BMC Pregnancy Childbirth: 94.8% (95% CI 93.8-95.8) | 90-96% | 40-100% |
| **Probability of presenting to testing** | | | |
| Base case: 100%  Clinical Scenario: 95% & 73% | Best case: WHO report from 2015 estimates 95% of infants receive a virological test by two months of age in South Africa  Worst case: Sherman et al 2015 found 73% of infants presented for EID at birth  Dunning et al (prepublication) 78% of HIV-exposed infants presented for EID | 73-95% | 0-100% |
| **Probability of result return** | | | |
| Base case: 100%  Clinical Scenario:80% | Ciaranello et al. 2011 | 57-80% | 0-100% |
| **Probability of linkage to HIV care** | | | |
| Base case: 100%  Clinical Scenario: 71% | Hsiao et al 2013 – 71% of infants who tested positive for HIV linked to care  Ciaranello et al 2011 – Best case: 81% Tanzania 2010  Worst case: 30% Malawi 2011 | 30-81% | 0-100% |

**Supplemental Table 2, continued.**

| **Point Estimate** | **Sources** | **Uncertainty Analysis Range** | **Sensitivity Analysis**  **Range** |
| --- | --- | --- | --- |
| **Mother-to-child transmission risks** | | | |
| ***In utero*/*intra partum* transmission, no ARVs** | | | |
| (CD4<350)  27.3% | Reviews of PMTCT trials:  Chigwedere *AIDS Res Hum Retrovir* 2008 meta-analysis (all CD4: 21%), Coutsoudis 2001 (19.9%), Nduati JAMA 1999 (19.9%), Fawzi JAIDS 2002 (15.4%).  One-time risk of MTCT stratified by CD4 based on the ZVITAMBO cohort (36%: Iliff, *AIDS* 2005)  Worst-case from literature: Leroy *AIDS* 2002 (all CD4: 25%)  Best-case from literature: Petra D (all CD4: 15%) | 15-25% (all CD4) | 6.8-60% |
| CD4 >350:   17.4% | Women not receiving ART during pregnancy with CD4>350 were assigned a one-time risk of 17.4%. Sources and calculations as above. | 15-25% (all CD4) | 4.3-35% |
| **Post-partum transmission, no ARVs** | | | |
| CD4 <350  Exclusive BF: 0.76%/m  Mixed BF: 1.28%/m | Monthly probability of MTCT risk was stratified by CD4 and breastfeeding status: One-time risk of MTCT stratified by CD4 based on the ZVITAMBO cohort (36%: Iliff, *AIDS* 2005)  EBF: Iliff *AIDS* 2005 (0.76%/m)  MBF: Iliff *AIDS* 2005 (1.28%/m)  Literature review found:  Best-case: Leroy *Lancet* 98 (0.5%/m)  Worst-case: Coutsoudis (2.3%/m) | 0.5-2.3%/m | EBF: 0.19-1.9%  MBF: 0.32-3.2% |
| CD4 >350  Exclusive BF: 0.24%/m  Mixed BF: 0.40%/m | As above.  CEPAC excluded the Tonwe-Gold study (due to difficulty determining accurate denominator for breastfeeding risk in primarily formula fed population) | 0.15-0.73%/m | EBF: 0.06-0.6%  MBF: 0.1-1.0% |
| ***In utero*/*intra partum* transmission, receiving ART in pregnancy** | | | |
| All CD4: 1.0% | Varying CD4 stratification of participants in PMTCT trials does not allow for formal meta-analysis – Review of literature:  MTCT Risk MCH-ART (all CD4): 1.7%  Kesho Bora (CD4 200-500): 3.3%  Mma Bana (CD4 <200, observational group): 0.6%  Mma Bana (CD4>200, 3 NRTI arm): 1.1%  Mma Bana (CD4 >200, PI arm): 0.4% [best case, >350]  DREAM (all CD4): 1.2%  KiBS (all CD4): 3.9% [worst case]  AMATA (all CD4): 1.43%  KiBS (CD4 >250): 3.8%  MASHI (all CD4): 2.8% | 0.4-3.9% | 0.25-4.0% |
| **Post-partum transmission, on ART** | | | |
| All CD4: 0.19%/m | As above, monthly risk of MTCT: Base case = mean of all except highest and lowest risks (0.19%)  Mma Bana (CD4 <200, observational): 0%/m  Mma Bana (CD4 >200, PI arm): 0%/m  Mma Bana (CD4 >200, 3 NRTI arm): 0.14%/m  BAN (CD4 >250): 0.53%/m  KiBS (CD4 >250): 0.23%/m  Mitra Plus (CD4 >200) : 0.25%/m  AMATA (all CD4): 0.10%/m  DREAM (all CD4): 0.20%/m | 0-0.53%/m | 0.05-0.5% |

**Table 2, continued.**

| **NAAT Characteristics** | | | |
| --- | --- | --- | --- |
| **Sensitivity of HIV assay** | | | |
| 1m after infection 100% | Mallampati D, Ford N, Hanaford A, Sugandhi N, Penazzato M. Performance of virological testing for early infant diagnosis: A systematic review. J Acquir Immune Defic Syndr. 2017;160(2012):1. | 98.3-100% | 90-100% |
| **Specificity of HIV assay** | | | |
| 99.6% | Mallampati D, Ford N, Hanaford A, Sugandhi N, Penazzato M. Performance of virological testing for early infant diagnosis: A systematic review. J Acquir Immune Defic Syndr. 2017;160(2012):1.  Previous meta-analysis found a reduced specificity of 98.8% | 99.1-100% | 90-100% |
| **ART** | | | |
| **ART efficacy by age, %** | | | |
| 0-59m 1^st^ line – 91%  2^nd^ line – 75% | Violari et al. N Eng J Med 2012  Palumbo et al. N Eng J Med 2010:  Babiker et al. Lancet ID 2011  Bath et al. Lancet ID 2010 (systematic review) found 82% pIS for both PI and NNRTI. | 91-82%  82-75% | n/a |
| >60m |  |  |  |
| **ART associated CD4 T-cell count-independent risk reduction, %** | | | |
| **Incidence of Opportunistic Infection, by age** | | | |
| 0-13m  85 | Ciaranello et al. AIDS 2015 - Cost-effectiveness of first-line antiretroviral therapy for HIV-infected African children less than three years of age. | 85-95% | 0-95% |
| >13m  32 | Losina et al 2007: a reduction in risks of OIs and death reported for patients on ART, regardless of whether ART is suppressive and in addition to the reduction in risk conferred by improvements in CD4 count alone | 2-50% | 0-90% |
| **Mortality, by age** | | | |
| 0-13m  90 | Ciaranello et al. AIDS 2015 - Cost-effectiveness of first-line antiretroviral therapy for HIV-infected African children less than three years of age. | 90-95% | 0-95% |
| >13m  55-96 | Losina et al 2007: a reduction in risks of OIs and death reported for patients on ART, regardless of whether ART is suppressive and in addition to the reduction in risk conferred by improvements in CD4 count alone | 57-96% | 0-96% |
| **Costs** | | | |
| **OI care, per event (range by age, CD4 T-cell %/count, type of event)** | | | |
| US $ 260-2,175 | Holmes C, Wood R, Badri M. CD4 decline and incidence of opportunistic infections in Cape Town, South Africa: Implications for prophylaxis and treatment. J Acquir Immune Defic Syndr. 2006;42:464–9.  Cleary S, Okorafor OA, Chitha W, Boulle A, Jikwana S. Financing antiretroviral treatment and primary health care services. South African Heal Rev. 2005;58–74. | n/a | $130-4350 |
| **ART, per month (regime/dose by age)** | | | |
| US $ 7-40 | Clinton Health Access Initiative. 2016 Antiretroviral (ARV) CHAI reference price list. 2016;(November):2015–6. Available from: http://www.clintonhealthaccess.org/content/uploads/2016/11/2016-CHAI-ARV- | n/a | $3-80 |
| **NAAT cost** | | | |
| Completed assay $25 | NHLS South Africa – Personal Communication | Assumption | $10-$400 |
| Result return for NAAT | Bassett I V, Giddy J, Nkera J, Wang B, Losina E, Lu Z, et al. Routine voluntary HIV testing in Durban, South Africa: the experience from an outpatient department. J Acquir Immune Defic Syndr. 2007;46(2):181–6 | n/a |  |

**EXTENDED RESULTS

Table 3. Base case Results with NAAT Specificity of 98.8%**

| **Model-based projections of the impact of false positive early infant diagnosis results in South Africa** | | | | |  |
| --- | --- | --- | --- | --- | --- |
|  | **Life expectancy (HIV-infected infants, years)** | **Number of False Positive infants initiating ART per 1000 ART initiations** | **Proportion of total lifetime costs due to care for infants with false positive diagnoses** | **Lifetime cost per HIV-exposed infant ($)^a^** |  |
| 6 week EID with confirmatory testing | 26.2 | 6 | 0.1% | $1790 |  |
| 6 week EID without confirmatory testing **^c^** | 26.2 | 298 | 6.0% | $1900 |  |
| **ICER**: incremental cost-effectiveness ratio; **N/A:** not applicable  a. Costs are in 2013 US dollars ($) and are undiscounted. b. We simulate ART initiation after the first positive EID assay result is received, with ART cessation if a confirmatory assay is subsequently negative. Because HIV-infected infants do not delay ART initiation for a confirmatory test result, the projected life expectancy for both EID strategies is similar. | | | | |  |

**Table 4. Model-based projections of the impact of false positive early infant diagnosis results in South Africa: sensitivity analyses, variation of key parameters**

| **Model-based projections of the impact of false positive early infant diagnosis results in South Africa: sensitivity analyses, variation of key parameters** | | | | | |
| --- | --- | --- | --- | --- | --- |
| Timing and frequency of NAAT | | | | | |
| **EID strategy** | **Life expectancy (HIV-infected infants, years)** | **Number of False Positive infants initiating ART per 1000 ART initiations** | **Proportion of total lifetime costs due to care for infants with false positive diagnoses** | **Lifetime cost per HIV-exposed infant ($)^a^** | |
| **With Confirmatory Testing** |  | |  | | |
| *No EID* | 20.9 | - | - | $1420 | |
| *Birth* | 24.5 | 1 | 0.01% | $1705 | |
| *6 weeks* | 26.2 | 1 | 0.01% | $1790 | |
| *Birth and 6 weeks* | 26.8 | 1 | 0.02% | $1850 | |
| **Without Confirmatory Testing** | | | | |  |
| *No EID* | 20.9 | - | - | $1420 | |
| *Birth* | 24.5 | 189 | 2.0% | $1680 | |
| *6 weeks* | 26.2 | 128 | 2.1% | $1830 | |
| *Birth and 6 weeks* | 26.8 | 213 | 4.0% | $1930 | |
| Presentation to EID testing | | | | | |
| **Probability of presenting to EID testing = 15%** | | | | | |
| *6 weeks with confirmatory testing* | 21.8 | 1 | 0.001% | $1470 | |
| *6 weeks without confirmatory testing* | 21.8 | 128 | 0.4% | $1480 | |
| **Probability of presenting to EID testing = 20%** | | | | | |
| *6 weeks with confirmatory testing* | 22.1 | 1 | 0.002% | $1490 | |
| *6 weeks without confirmatory testing* | 22.1 | 128 | 0.5% | $1500 | |
| **Probability of presenting to EID testing = 30%** | | | | | |
| *6 weeks with confirmatory testing* | 22.6 | 1 | 0.003% | $1530 | |
| *6 weeks without confirmatory testing* | 22.6 | 128 | 0.7% | $1540 | |
| **Probability of presenting to EID testing = 40%** | | | | | |
| *6 weeks with confirmatory testing* | 23.1 | 1 | 0.004% | $1560 | |
| *6 weeks without confirmatory testing* | 23.1 | 128 | 0.9% | $1585 | |
| **Probability of presenting to EID testing = 50%** | | | | | |
| *6 weeks with confirmatory testing* | 23.6 | 1 | 0.004% | $1605 | |
| *6 weeks without confirmatory testing* | 23.6 | 128 | 1.2% | $1625 | |

**Table 4 (continued)**

| **EID strategy** | **Life expectancy (HIV-infected infants, years)** | **Number of False Positive infants initiating ART per 1000 ART initiations** | **Proportion of total lifetime costs due to care for infants with false positive diagnoses** | **Lifetime cost per HIV-exposed infant ($)^a^** |
| --- | --- | --- | --- | --- |
| Result Return | | | | |
| **Probability of EID result being returned to caregiver = 10%** | | | | |
| *6 weeks with confirmatory testing* | 21.5 | 1 | 0.0001% | $1480 |
| *6 weeks without confirmatory testing* | 21.5 | 128 | 0.3% | $1485 |
| **Probability of EID result being returned to caregiver = 20%** | | | | |
| *6 weeks with confirmatory testing* | 22.1 | 1 | 0.0002% | $1515 |
| *6 weeks without confirmatory testing* | 22.1 | 128 | 0.5% | $1520 |
| **Probability of EID result being returned to caregiver = 30%** | | | | |
| *6 weeks with confirmatory testing* | 22.6 | 1 | 0.001% | $1550 |
| *6 weeks without confirmatory testing* | 22.6 | 128 | 0.7% | $1560 |
| **Probability of EID result being returned to caregiver = 40%** | | | | |
| *6 weeks with confirmatory testing* | 23.1 | 1 | 0.002% | $1580 |
| *6 weeks without confirmatory testing* | 23.1 | 128 | 0.9% | $1595 |
| **Probability of EID result being returned to caregiver = 50%** | | | | |
| *6 weeks with confirmatory testing* | 23.6 | 1 | 0.002% | $1620 |
| *6 weeks without confirmatory testing* | 23.6 | 128 | 1.2% | $1640 |
| Linkage to care | | | | |
| **Probability of linkage to care and ART after confirmed HIV diagnosis = 15%** | | | | |
| *6 weeks with confirmatory testing* | 21.8 | 1 | 0.001% | $1505 |
| *6 weeks without confirmatory testing* | 21.8 | 128 | 0.3% | $1510 |
| **Probability of linkage to care and ART after confirmed HIV diagnosis = 20%** | | | | |
| *6 weeks with confirmatory testing* | 22.0 | 1 | 0.002% | $1525 |
| *6 weeks without confirmatory testing* | 22.0 | 128 | 0.5% | $1530 |
| **Probability of linkage to care and ART after confirmed HIV diagnosis = 30%** | | | | |
| *6 weeks with confirmatory testing* | 22.6 | 1 | 0.003% | $1560 |
| *6 weeks without confirmatory testing* | 22.6 | 128 | 0.7% | $1570 |
| **Probability of linkage to care and ART after confirmed HIV diagnosis = 40%** | | | | |
| *6 weeks with confirmatory testing* | 23.1 | 1 | 0.004% | $1590 |
| *6 weeks without confirmatory testing* | 23.1 | 128 | 0.9% | $1605 |

**Table 4 (continued)**

| **Probability of linkage to care and ART after confirmed HIV diagnosis = 50%** | | | | | | | | |
| --- | --- | --- | --- | --- | --- | --- | --- | --- |
| *6 weeks with confirmatory testing* | | 23.6 | 1 | | 0.005% | | $1620 | |
| *6 weeks without confirmatory testing* | | 23.6 | 128 | | 1.2% | | $1640 | |
| Specificity of NAAT assay | | | | | | | | |
| **Specificity of NAAT assay 90.0%** | | | | | | | | |
| *6 weeks with confirmatory testing* | | 26.2 | 295 | | 5.1% | | $1890 | |
| *6 weeks without confirmatory testing* | | 26.2 | 784 | | 34.6% | | $2740 | |
| **Specificity of NAAT assay 92.0%** | | | | | | | | |
| *6 weeks with confirmatory testing* | | 26.2 | 210 | | 3.3% | | $1850 | |
| *6 weeks without confirmatory testing* | | 26.2 | 745 | | 29.8% | | $2550 | |
| **Specificity of NAAT assay 94.0%** | | | | | | | | |
| *6 weeks with confirmatory testing* | | 26.2 | 130 | | 1.9% | | $1830 | |
| *6 weeks without confirmatory testing* | | 26.2 | 687 | | 24.1% | | $2360 | |
| **Specificity of NAAT assay 96.0%** | | | | | | | | |
| *6 weeks with confirmatory testing* | | 26.2 | 1 | | 0.01% | | $1770 | |
| *6 weeks without confirmatory testing* | | 26.2 | 132 | | 2.1% | | $1810 | |
| **Specificity of NAAT assay 98.0%** | | | | | | | | |
| *6 weeks with confirmatory testing* | | 26.2 | 1 | | 0.01% | | $1780 | |
| *6 weeks without confirmatory testing* | | 26.2 | 130 | | 2.1% | | $1820 | |
| **Specificity of NAAT assay 99.0%** | | | | | | | | |
| *6 weeks with confirmatory testing* | | 26.2 | 4 | | 0.05% | | $1790 | |
| *6 weeks without confirmatory testing* | | 26.2 | 260 | | 5.0% | | $1890 | |
| **Specificity of NAAT assay 99.2%** | | | | | | | | |
| *6 weeks with confirmatory testing* | 26.2 | | | 3 | | 0.03% | | $1790 |
| *6 weeks without confirmatory testing* | 26.2 | | | 219 | | 4.1% | | $1870 |
| **Specificity of NAAT assay 99.5%** | | | | | | | | |
| *6 weeks with confirmatory testing* | 26.2 | | | 2 | | 0.02% | | $1790 |
| *6 weeks without confirmatory testing* | 26.2 | | | 174 | | 3.1% | | $1850 |
| **Specificity of NAAT assay 99.8%** | | | | | | | | |
| *6 weeks with confirmatory testing* | 26.2 | | | 0 | | 0.01% | | $1790 |
| *6 weeks without confirmatory testing* | 26.2 | | | 66 | | 1.0% | | $1810 |

**Table 4 (continued)**

| Mother To Child Transmission (MTCT) rate / Infant HIV prevalence | | | | | | | | | |
| --- | --- | --- | --- | --- | --- | --- | --- | --- | --- |
| **Total MTCT: 1.26% (Early MTCT: 0.74%)** | | | | | | | | | |
| *6 weeks with confirmatory testing* | | 26.2 | 3 | | | | 0.03% | | $480 |
| *6 weeks without confirmatory testing* | | 26.2 | 375 | | | | 7.5% | | $520 |
| **Total MTCT: 2.5% (Early MTCT: 1.5%)** | | | | | | | | | |
| *6 weeks with confirmatory testing* | | 26.2 | 2 | | | | 0.02% | | $920 |
| *6 weeks without confirmatory testing* | | 26.2 | 229 | | | | 4.0% | | $960 |
| **Total MTCT: 7.2% (Early MTCT: 4.4%)** | | | | | | | | | |
| *6 weeks with confirmatory testing* | | 26.2 | 0 | | | | 0.01% | | $2630 |
| *6 weeks without confirmatory testing* | | 26.2 | 88 | | | | 1.4% | | $2660 |
| **Total MTCT: 9.5% (Early MTCT: 5.9%)** | | | | | | | | | |
| *6 weeks with confirmatory testing* | | 26.2 | 0 | | | | 0.004% | | $3440 |
| *6 weeks without confirmatory testing* | | 26.2 | 66 | | | | 1.1% | | $3470 |
| **Total MTCT: 11.7% (Early MTCT: 7.4%)** | | | | | | | | | |
| *6 weeks with confirmatory testing* | | 26.2 | 0 | | | | 0.003% | | $4220 |
| *6 weeks without confirmatory testing* | | 26.2 | 53 | | | | 0.8% | | $4250 |
| Based on preliminary data about emerging Point-of-Care (POC) assays | | | | | | | | | |
| **95.5% sensitivity, 99.8% specificity, POC cost per assay $30** | | | | | | | | | |
| *6 weeks with confirmatory testing* | 25.9 | | | | 0 | 0.002% | | $1730 | |
| *6 weeks without confirmatory testing* | 25.9 | | | | 69 | 1.1% | | $1790 | |
| Sensitivity of NAAT assay | | | | | | | | | |
| **Sensitivity of NAAT assay 96.0%** | | | | | | | | | |
| *6 weeks with confirmatory testing* | 25.7 ^a^ | | | | 1 | 0.01% | | $1770 | |
| *6 weeks without confirmatory testing* | 26.0 | | | | 132 | 2.1% | | $1810 | |
| **Sensitivity of NAAT assay 99.0%** | | | | | | | | | |
| *6 weeks with confirmatory testing* | 26.1 ^a^ | | | 1 | | 0.01% | | $1785 | |
| *6 weeks without confirmatory testing* | 26.2 | | | 128 | | 2.1% | | $1825 | |
| **Sensitivity of NAAT assay 99.5%** | | | | | |  | | | |
| *6 weeks with confirmatory testing* | 26.2 | | 1 | | | 0.01% | | $1790 | |
| *6 weeks without confirmatory testing* | 26.2 | | 128 | | | 2.1% | | $1825 | |
| a Difference in LE between the *without and with confirmatory testing* strategies are the result of fewer infected infants remaining on ART when one confirmatory assay is used. When two confirmatory tests are used for infants with discordant results (as per WHO guidelines) LE remains equal between the two strategies; *with confirmatory testing* remains cost saving | | | | | | | | | |

**Table 4 (continued)**

| Mother To Child Transmission (MTCT) rate / Infant HIV prevalence | | | | |
| --- | --- | --- | --- | --- |
| **Total MTCT: 2.5% (Early MTCT: 1.5%)** | | | | |
| *6 weeks with confirmatory testing* | 26.2 | 1 | 0.01% | $925 |
| *6 weeks without confirmatory testing* | 26.2 | 228 | 4.0% | $960 |
| **Total MTCT: 6.1% (Early MTCT: 3.7%)** | | | | |
| *6 weeks with confirmatory testing* | 26.3 | 1 | 0.01% | $2215 |
| *6 weeks without confirmatory testing* | 26.3 | 105 | 1.4% | $2250 |
| **Total MTCT: 7.2% (Early MTCT: 4.4%)** | | | | |
| *6 weeks with confirmatory testing* | 26.3 | 0 | 0.01% | $2630 |
| *6 weeks without confirmatory testing* | 26.3 | 88 | 1.4% | $2660 |
| **Total MTCT: 8.4% (Early MTCT: 5.2%)** | | | | |
| *6 weeks with confirmatory testing* | 26.3 | 0 | 0.004% | $3040 |
| *6 weeks without confirmatory testing* | 26.3 | 75 | 1.2% | $3070 |
| **Total MTCT: 9.6% (Early MTCT: 5.7%)** | | | | |
| *6 weeks with confirmatory testing* | 24.4 | 0 | 0.003% | $3400 |
| *6 weeks without confirmatory testing* | 25.8 | 66 | 1.1% | $3470 |
| Result return time | | | | |
| **Result return time = 0 months** | | | | |
| *6 weeks with confirmatory testing* | 27.3 | 1 | 0.01% | $1855 |
| *6 weeks without confirmatory testing* | 27.3 | 127 | 2.0% | $1895 |
| **Result return time = 2 months** | | | | |
| *6 weeks with confirmatory testing* | 25.3 | 1 | 0.01% | $1730 |
| *6 weeks without confirmatory testing* | 25.3 | 128 | 2.1% | $1765 |
| **Result return time = 3 months** | | | | |
| *6 weeks with confirmatory testing* | 24.4 | 1 | 0.1% | $1670 |
| *6 weeks without confirmatory testing* | 24.4 | 128 | 2.2% | $1705 |
| **Result return time = 4 months** | | | | |
| *6 weeks with confirmatory testing* | 23.7 | 1 | 0.01% | $1625 |
| *6 weeks without confirmatory testing* | 23.7 | 128 | 2.3% | $1660 |

**Table 4 (continued)**

| Examining conditional independence of the first and confirmatory assays | | | | |
| --- | --- | --- | --- | --- |
| **0.0% Specificity of confirmatory NAAT** | | | | |
| *6 weeks with confirmatory testing* | 26.2 | 128 | 2.1% | $1830 |
| **10.0% Specificity of confirmatory NAAT** | | | | |
| *6 weeks with confirmatory testing* | 26.2 | 115 | 1.9% | $1825 |
| **15.0% Specificity of confirmatory NAAT** | | | | |
| *6 weeks with confirmatory testing* | 26.2 | 110 | 1.8% | $1825 |
| **20.0% Specificity of confirmatory NAAT** | | | | |
| *6 weeks with confirmatory testing* | 26.2 | 104 | 1.7% | $1820 |
| **40.0% Specificity of confirmatory NAAT** | | | | |
| *6 weeks with confirmatory testing* | 26.2 | 80 | 1.3% | $1810 |
| **60.0% Specificity of confirmatory NAAT** | | | | |
| *6 weeks with confirmatory testing* | 26.2 | 55 | 0.8% | $1805 |
| **70.0% Specificity of confirmatory NAAT** | | | | |
| *6 weeks with confirmatory testing* | 26.2 | 41 | 0.6% | $1800 |
| **80.0% Specificity of confirmatory NAAT** | | | | |
| *6 weeks with confirmatory testing* | 26.2 | 28 | 8.4% | $1800 |
| **90.0% Specificity of confirmatory NAAT** | | | | |
| *6 weeks with confirmatory testing* | 26.2 | 15 | 0.2% | $1795 |
| **95.0% Specificity of confirmatory NAAT** | | | | |
| *6 weeks with confirmatory testing* | 26.2 | 7 | 0.1% | $1790 |
| **99.0% Specificity of confirmatory NAAT** | | | | |
| *6 weeks with confirmatory testing* | 26.2 | 1 | 0.02% | $1790 |
| **99.9% Specificity of confirmatory NAAT** | | | | |
| *6 weeks with confirmatory testing* | 26.2 | 0 | 0.002% | $1790 |
| Delayed initiation of ART until return of confirmatory test | | | | |
| **ART initiated on return of confirmatory test** | | | | |
| *6 weeks with confirmatory testing* | 25.8 | 1 | 0.01% | $1790 |
| *6 weeks without confirmatory testing* | 26.2 | 128 | 2.1% | $1830 |

(Abbreviations: **ART:** Antiretroviral therapy, **NAAT:** Nucleic Acid Amplification Test, **EID:** Early infant diagnosis, **MTCT:** mother-to-child transmission)

**Table 5:** **Model-based projections of the impact of false-positive early infant diagnosis results in South Africa with and without confirmatory testing:** **alterations to costs**

| **EID strategy** | **Life expectancy (HIV-infected infants, years)** | **Proportion of total lifetime costs due to care for infants with false positive diagnoses** | **Lifetime cost per HIV-exposed infant ($)^a^** |
| --- | --- | --- | --- |
| Cost of TNA assay | | | |
| **NAAT assay cost of $40** | | | |
| *6 weeks with confirmatory testing* | 26.2 | 0.01% | $1805 |
| *6 weeks without confirmatory testing* | 26.2 | 2.1% | $1835 |
| **NAAT assay cost of $50** | | | |
| *6 weeks with confirmatory testing* | 26.2 | 0.01% | $1815 |
| *6 weeks without confirmatory testing* | 26.2 | 2.0% | $1850 |
| **NAAT assay cost of $60** | | | |
| *6 weeks with confirmatory testing* | 26.2 | 0.01% | $1825 |
| *6 weeks without confirmatory testing* | 26.2 | 2.0% | $1860 |
| **NAAT assay cost of $80** | | | |
| *6 weeks with confirmatory testing* | 26.2 | 0.01% | $1850 |
| *6 weeks without confirmatory testing* | 26.2 | 2.0% | $1890 |
| **NAAT assay cost of $150** | | | |
| *6 weeks with confirmatory testing* | 26.2 | 0.01% | $1920 |
| *6 weeks without confirmatory testing* | 26.2 | 1.9% | $1950 |
| **NAAT assay cost of $200** | | | |
| *6 weeks with confirmatory testing* | 26.2 | 0.01% | $2000 |
| *6 weeks without confirmatory testing* | 26.2 | 1.9% | $2015 |
| **NAAT assay cost of $300** | | | |
| *6 weeks with confirmatory testing* | 26.2 | 0.01% | $2105 |
| *6 weeks without confirmatory testing* | 26.2 | 1.8% | $2115 |
| **NAAT assay cost of $400** | | | |
| *6 weeks with confirmatory testing* | 26.2 | 0.01% | $2210 |
| *6 weeks without confirmatory testing* | 26.2 | 1.7% | $2210 |
| **NAAT assay cost of $500** | | | |
| *6 weeks with confirmatory testing* | 26.2 | 0.01% | $2310 |
| *6 weeks without confirmatory testing* | 26.2 | 1.6% | $2310 |

**Table 5 (continued)**

| Variations of clinical care costs | | | |
| --- | --- | --- | --- |
| **Clinical care costs x 0.5** |  |  |  |
| *6 weeks with confirmatory testing* | 26.2 | 0.01% | $1090 |
| *6 weeks without confirmatory testing* | 26.2 | 2.0% | $1110 |
| **Clinical care costs x 2.0** |  |  |  |
| *6 weeks with confirmatory testing* | 26.2 | 0.01% | $3190 |
| *6 weeks without confirmatory testing* | 26.2 | 2.0% | $3265 |
| Variations of ART care costs | | | |
| **ART costs x 0.5** |  |  |  |
| *6 weeks with confirmatory testing* | 26.2 | 0.01% | $1655 |
| *6 weeks without confirmatory testing* | 26.2 | 2.0% | $1690 |
| **ART costs x 1.5** |  |  |  |
| *6 weeks with confirmatory testing* | 26.2 | 0.01% | $1925 |
| *6 weeks without confirmatory testing* | 26.2 | 2.1% | $1970 |
| **ART costs x 2.0** |  |  |  |
| *6 weeks with confirmatory testing* | 26.2 | 0.01% | $2060 |
| *6 weeks without confirmatory testing* | 26.2 | 2.2% | $2110 |
| OI costs | | | |
| **No OI costs** | | | |
| *6 weeks with confirmatory testing* | 26.2 | 0.1% | $1335 |
| *6 weeks without confirmatory testing* | 26.2 | 2.8% | $1375 |

(Abbreviations: **ART:** Antiretroviral therapy **NAAT:** Nucleic Acid Amplification Test

**Table 6:** **Multivariate analyses, varying MTCT risk and specificity of NAAT assay: Model-based projections of the impact of false-positive early infant diagnosis results in South Africa with and without confirmatory testing**

|  | T**ransmission Risks** | | |  | |  | |  | |  |  |  |
| --- | --- | --- | --- | --- | --- | --- | --- | --- | --- | --- | --- | --- |
|  | 0.25x  (1.3% MTCT) | | 0.5x  (2.5% MTCT) | | 1.25x   (6.1% MTCT) | | 1.5x   (7.2% MTCT) | | 1.75x  (8.4% MTCT) | | 2x  (9.6% MTCT) | |
| **Specificity** | With Conf testing | Without Conf testing | With Conf. testing | Without Conf testing | With Conf testing | Without Conf testing | With Conf testing | Without Conf testing | With Conf testing | Without Conf testing | With Conf testing | Without Conf testing |
| Specificity 98.0% | | | | | | | | | | | | |
| **False-positives per 1000 ART initiations** | 63 | 749 | 33 | 597 | 13 | 367 | 11 | 324 | 9 | 290 | 8 | 262 |
| **Lifetime cost per HIV-exposed infant ($)** | $480 | $670 | $930 | $1120 | $2220 | $2400 | $2635 | $2815 | $3040 | $3220 | $3450 | $3620 |
| Specificity 98.2% | | | | | | | | | | | | |
| **False-positives per 1000 ART initiations** | 52 | 729 | 26 | 571 | 11 | 342 | 9 | 301 | 7 | 269 | 7 | 241 |
| **Lifetime cost per HIV-exposed infant ($)** | $480 | $655 | $930 | $1100 | $2220 | $2380 | $2635 | $2800 | $3040 | $3200 | $3440 | $3600 |
| Specificity 98.8% | | | | | | | | | | | | |
| **False-positives per 1000 ART initiations** | 24 | 641 | 12 | 471 | 5 | 258 | 4 | 224 | 3 | 196 | 3 | 175 |
| **Lifetime cost per HIV-exposed infant ($)** | $480 | $600 | $925 | $1040 | $2215 | $2325 | $2630 | $2735 | $3040 | $3140 | $3440 | $3540 |
| Specificity 99.0% | | | | | | | | | | | | |
| **False-positives per 1000 ART initiations** | 17 | 599 | 8 | 426 | 3 | 224 | 3 | 193 | 2 | 170 | 2 | 150 |
| **Lifetime cost per HIV-exposed infant ($)** | $480 | $575 | $925 | $1020 | $2215 | $2305 | $2630 | $2720 | $3040 | $3125 | $3440 | $3525 |

| **Table 6 (continued)** | T**ransmission Risks** | | | | | | | | | | | |
| --- | --- | --- | --- | --- | --- | --- | --- | --- | --- | --- | --- | --- |
|  | 0.25x  (1.3% MTCT) | | 0.5x  (2.5% MTCT) | | 1.25x   (6.1% MTCT) | | 1.5x   (7.2% MTCT) | | 1.75x  (8.4% MTCT) | | 2x  (9.6% MTCT) | |
| **Specificity** | With Conf testing | Without Conf testing | With Conf. testing | Without Conf testing | With Conf testing | Without Conf testing | With Conf testing | Without Conf testing | With Conf testing | Without Conf testing | With Conf testing | Without Conf testing |
| Specificity 99.2% |  |  |  |  |  |  |  |  |  |  |  |  |
| **False-positives per 1000 ART initiations** | 11 | 544 | 5 | 372 | 2 | 189 | 2 | 161 | 1 | 140 | 1 | 124 |
| **Lifetime cost per HIV-exposed infant ($)** | $480 | $560 | $925 | $1000 | $2215 | $2290 | $2630 | $2700 | $3040 | $3110 | $3440 | $3510 |
| Specificity 99.5% | | | | | | | | | | | | |
| **False-positives per 1000 ART initiations** | 4 | 429 | 2 | 270 | 1 | 127 | 1 | 107 | 1 | 92 | 0 | 82 |
| **Lifetime cost per HIV-exposed infant ($)** | $480 | $530 | $925 | $970 | $2215 | $2260 | $2630 | $2670 | $3040 | $3080 | $3440 | $3480 |
| Specificity 99.6% | | | | | | | | | | | | |
| **False-positives per 1000 ART initiations** | 3 | 373 | 1 | 228 | 1 | 105 | 0 | 88 | 0 | 75 | 0 | 66 |
| **Lifetime cost per HIV-exposed infant ($)** | $480 | $520 | $925 | $960 | $2215 | $2250 | $2630 | $2660 | $3040 | $3070 | $3440 | $3470 |
| Specificity 99.8% | | | | | | | | | | | | |
| **False-positives per 1000 ART initiations** | 1 | 229 | 0 | 129 | 0 | 54 | 0 | 46 | 0 | 39 | 0 | 34 |
| **Lifetime cost per HIV-exposed infant ($)** | $480 | $500 | $925 | $945 | $2215 | $2230 | $2630 | $2640 | $3040 | $3060 | $3440 | $3450 |

Abbreviations: **ART:** Antiretroviral therapy **Conf:** Confirmatory **MTCT:** Mother-to-Child Transmission **NAAT:** Nucleic Acid Amplification Test

**Table 7:** **Multivariate analyses, varying infant HIV prevalence, specificity of NAAT assay and clinical health care costs: Model-based projections of the impact of false-positive early infant diagnosis results in South Africa with and without confirmatory testing**

| Infant HIV prevalence / specificity of NAAT / clinical care costs | | | | |
| --- | --- | --- | --- | --- |
| **1.5x MTCT risk, 99.8% specificity, 0.5x clinical care costs** | | | | |
| *6 weeks with confirmatory testing* | 26.2 | 0 | 0.001% | $1935 |
| *6 weeks without confirmatory testing* | 26.2 | 46 | 0.6% | $1945 |
| **2x MTCT risk, 99.8% specificity, 0.5x clinical care costs** | | | | |
| *6 weeks with confirmatory testing* | 26.2 | 0 | 0.001% | $2525 |
| *6 weeks without confirmatory testing* | 26.2 | 34 | 0.5% | $2535 |
| **2x MTCT risk, 99.8% specificity, 2x clinical care costs** | | | | |
| *6 weeks with confirmatory testing* | 26.2 | 0 | 0.001% | $4350 |
| *6 weeks without confirmatory testing* | 26.2 | 34 | 0.6% | $4360 |
| **1.5x MTCT risk, 99.8% specificity, 2x clinical care costs** | | | | |
| *6 weeks with confirmatory testing* | 26.2 | 0 | 0.004% | $4020 |
| *6 weeks without confirmatory testing* | 26.2 | 46 | 1.4% | $4070 |
| **NAAT assay cost $30, 99.8% specificity, 2x clinical care costs** | | | | |
| *6 weeks with confirmatory testing* | 26.2 | 0 | 0.004% | $4020 |
| *6 weeks without confirmatory testing* | 26.2 | 46 | 1.4% | $4070 |

Without confirmatory testing

With confirmatory testing

**Figure 1. Average lifetime costs per HIV-exposed infant by duration of HIV care and ART after a false positive diagnosis**.
Univariate sensitivity analysis varying time infants with a false positive diagnosis link to HIV and ART care, with and without confirmatory testing for 6 week EID visit. Left axis depicts the lifetime cost for HIV exposed infants. The horizontal axis is is the variation in time linked to care. Blue line demonstrates the variation in in lifetime cost for HIV exposed infants without confirmatory EID testing, red line indicates the lifetime cost for HIV exposed infants when confirmatory testing is used.

**Figure 2: Univariate sensitivity analysis varying assay NAAT specificity without and with confirmatory testing for both 6-week EID testing and Birth and 6-week testing algorithms.**Left axis depicts infants initiating ART with a false positive diaogisis per 1000 ART initiations. The horizontal axis is is the variation assay specficity. Blue lines in number of false-positive infants for each value of specificity without confirmatory EID testing, the red line indicates the number when confirmatory testing is used.

**Figure 3

Figure 3: Assessing structural uncertainty.**We repeated the comparison of *with* vs. *without confirmatory testing* using 25 parameter sets reflecting all combinations of the 5 best-fitting parameter sets from a calibration of survival among untreated children (table from *PLoS ONE* 2013 below; colors red, dark plue, purple, green, and light blue), the 5 best-fitting parameter sets from a calibration of survival among treated children (table from *AIDS* 2015 below; shapes circle, circle, rectangle, triangle, dash, and diamond). See Appendix text above for additional information. The base-case analysis uses the combination shown as a red diamond. The horizontal axis shows the cost savings over a lifetime per HIV-exposed infant between the *with confirmatory testing* strategy compared to the *without confirmatory testing* strategy (base-case difference, $40). With all 25 parameter sets, *with confirmatory testing* remained cost-saving compared to *without confirmatory testing*.

**Parameters included from the structural uncertainty assessment for untreated children are taken from Ciaranello AL, Morris BL, Walensky RP, Weinstein MC, Ayaya S, Doherty K, et al. Validation and calibration of a computer simulation model of pediatric HIV infection. PLoS One. 2013;8(12):e83389. (Table 5). Root-mean-squared error for key parameters sets in the calibration of the CEPAC-Pediatric model to UNAIDS survival data**

|  |  |  | **Chronic HIV mortality multiplier ^c^** | | | | | |  |
| --- | --- | --- | --- | --- | --- | --- | --- | --- | --- |
| **Mean CD4% at birth** | **Monthly CD4% decline ^a^** | **Acute clinical event mortality multiplier ^b^** | **0-6m** | **7-12m** | **13-24m** | **25-36m** | **37-48m** | **49-60m** | **Root-mean-squared error (RMSE) ^d^** |
| **10 best-fitting parameter sets** | | | | | | | | | |
| **45** | **4, 0.5** | **4** | **17** | **7** | **3** | **1.5** | **1.2** | **0.4** | **0.00122** |
| **42** | **3, 0.5** | **4** | **17** | **7** | **3** | **1.5** | **1.2** | **0.4** | **0.00146** |
| **50** | **4, 0.5** | **5** | **17** | **8** | **3.5** | **2** | **1.6** | **0.4** | **0.00152** |
| **47** | **3, 0.5** | **5** | **17** | **8** | **3.5** | **2** | **1.6** | **0.4** | **0.00162** |
| **50** | **4, 0.5** | **5** | **17** | **8** | **3.5** | **2** | **1.6** | **0.6** | **0.00172** |
| **Base-case IeDEA survival from internal validation analysis, projected to 60 months of age** | | | | | | | | | |
| 45 | 6, 0.3 | 1 | 1 | 1 | 1 | 1 | 1 | 1 | 0.383 |
| **Lowest-mortality risk parameter set from Table 3, projected to 60 months of age** | | | | | | | | | |
| 50 | 3, 0.3 | 0.5 | 1 | 1 | 0.5 | 0.5 | 0.2 | 0.2 | 0.575 |
| **Highest-mortality risk parameter set from Table 3, projected to 60 months of age** | | | | | | | | | |
| 42 | 8, 1.4 | 5 | 20 | 20 | 5 | 5 | 2 | 2 | 0.236 |

a. CD4% decline is shown as monthly decline (in CD4 percentage points) for months 1-3 of life, followed by for months 4+ of life.

b. Multipliers were applied to the monthly risks of "acute mortality" derived from the IeDEA cohort (defined as mortality <30 days following a WHO3, WHO4, or TB clinical event).

c. Multipliers were applied to monthly risks of "chronic HIV mortality" derived from the IeDEA cohort (defined separately as mortality risks among infants with no history of clinical event, or >30 days after a clinical event for infants with a history of clinical event).

d. Root-mean-squared error of CEPAC-Pediatric model projections compared to UNAIDS survival data at 6, 12, 24, 36, 48, and 60 months of age. RMSE is calculated by 1) calculating the difference between observed and projected survival proportions at each time point, 2) squaring these six absolute differences, 3) averaging the squared values, and 4) taking the square root of this average value. RMSE reflects an average difference between observed and projected survival (as a percent) over the six time points.

Parameters included from the structural uncertainty assessment for treated children are taken from Ciaranello *et al.,*  Cost-effectiveness of first-line antiretroviral therapy for HIV-infected African children less than three years of age: Supplemental Appendix AIDS. 2015; 29(10):1247–59, describing calibration of the CD4-independent impact of ART: Identification of multipliers producing closest fit to observed mortality for children presenting to care at 12 months of age, treated with the *first-line nevirapine* strategy

| **Relative risk reduction (for mortality; OI risk)** ^a^ | **Rate/100PY (2-year average)** | **Rate/100PY (4-year average)** |
| --- | --- | --- |
| P1060: observed mortality rate (comparator; median follow-up: 72-weeks) (base-case 90%, 85%) | 3.29 | |
| 100%, 100% | 2.57 | 1.87 |
| 95%, 95% | 3.38 | 2.62 |
| 90%, 95% | 3.98 | 3.02 |
| 95%, 90% | 4.04 | 3.23 |
| 90%, 90% | 4.67 | 3.65 |
| 85%, 90% | 5.34 | 4.25 |
| 90%, 75% | 6.62 | 5.40 |
| 75%, 85% | 7.28 | 5.54 |
| 75%, 75% | 8.59 | 6.74 |
| 50%, 85% | 10.56 | 7.73 |
| 90%, 50% | 9.66 | 8.13 |
| 25%, 85% | 13.82 | 9.87 |
| 90%, 25% | 12.54 | 10.71 |
| 50%, 50% | 15.25 | 11.76 |
| 0%, 85% | 17.14 | 12.04 |
| 90%, 0% | 15.24 | 13.19 |
| 25%, 25% | 21.59 | 16.52 |
| 0%, 0% | 27.72 | 21.07 |

Yellow highlighting indicates model projections that best fit mortality data from the P1060 trial (please see text).

1. Compared to children not on ART with similar CD4 (see text).

**REFERENCES**

1. Ciaranello AL, Doherty K, Penazzato M, Lindsey JC, Harrison L, Kelly K, et al. Cost-effectiveness of first-line antiretroviral therapy for HIV-infected African children less than 3 years of age. AIDS. 2015;29(10):1247–59.

2. Ciaranello AL, Morris BL, Walensky RP, Weinstein MC, Ayaya S, Doherty K, et al. Validation and calibration of a computer simulation model of pediatric HIV infection. PLoS One. 2013;8(12):1–13.

# 3. Ciaranello AL, Myer L, Kelly K, Christensen S, Daskilewicz K, Doherty K, et al. (2015) Point-of-Care CD4 Testing to Inform Selection of Antiretroviral Medications in South African Antenatal Clinics: A Cost-Effectiveness Analysis. PLoS ONE10(3): e0117751.

4. World Health Organization. Consolidated guidelines on the use of antiretroviral drugs for treating and preventing HIV infection: recommendations for a public health approach. Geneva, Switzerland; 2016. Available from: http://www.who.int/hiv/pub/guidelines/arv2013/download/en/index.html.

5. World Health Organization. Recommendations on the Diagnosis of HIV Infection in Infants and Children. WHO Guidelines. Geneva, Switzerland; 2010. Available from: http://apps.who.int/iris/bitstream/10665/44275/1/9789241599085_eng.pdf

6. Republic of South Africa - National Department of Health. National Consolidated Guidelines: For the prevention of mother-to-child transmission of HIV (PMTCT) and the management of HIV in children, adolescents and adults. 2015. Available from: https://aidsfree.usaid.gov/sites/default/files/tx_south-africa_pmtct_2015.pdf

7. Losina E, Yazdanpanah Y, Deuffic-Burban S, Wang B, Wolf LL, Messou E, et al. The independent effect of highly active antiretroviral therapy on severe opportunistic disease incidence and mortality in HIV-infected adults in Cote d’Ivoire. Antivir Ther. 2007;12(4):543–51.

8. Desmonde S, Frank SC, Coovadia A, Dahourou DL, Abrams EJ, Walensky RP, et al. Cost-effectiveness of pre-emptive switching to efavirenz ( EFV ) in HIV-infected children. In: Conference of Retroviruses and Opportunistic Infections. Seattle, USA; 2017. p. eposter: 1027.

9. Violari A, Lindsey JC, Hughes MD et al. Nevirapine versus Ritonavir-Boosted Lopinavir for HIV-Infected Children. N Engl J Med. 2012;366(25):2380–9.

10. Palumbo P, Lindsey J, Hughes M. Antiretroviral treatment for children with peripartum nevirapine exposure. N Engl J Med. 2010;363:1510–20.

11. Kilewo C, Karlsson K, Ngarina M, Massawe A, Lyamuya E, Swai A, et al. Prevention of Mother-to-Child Transmission of HIV-1 Through Breastfeeding by Treating Mothers With Triple Antiretroviral Therapy in Dar es Salaam, Tanzania: The Mitra Plus Study. JAIDS J Acquir Immune Defic Syndr. 2009;52(3):406–16.

12. Petra Study Team. Efficacy of three short-course regimens of zidovudine and lamivudine in preventing early and late transmission of HIV-1 from mother to child in Tanzania, South Africa, and Uganda (Petra study): A randomised, double-blind, placebo-controlled trial. Lancet. 2002;359(9313):1178–86.

13. Chigwedere P, Seage G, Lee T, Essex M. Efficacy of antiretroviral drugs in reducing mother-to-child transmission of HIV in Africa: a meta-analysis of published clinical trials. AIDS Res Hum Retroviruses. 2008;24(6):827–37.

14. Tonwe-Gold B, Ekouevi DK, Viho I, Amani-Bosse C, Toure S, Coffie PA, et al. Antiretroviral treatment and prevention of peripartum and postnatal HIV transmission in West Africa: Evaluation of a two-tiered approach. PLoS Med. 2007;4(8):1362–73.

15. Leroy V, Karon J, Alioum A, Ekpini E, Meda N, Greenberg A. Twenty-four month efficacy of a maternal short-course zidovudine regimen to prevent mother-to-child transmission of HIV-1 in West Africa. AIDS. 2002;16(4):631–41.

16. Mallampati D, Ford N, Hanaford A, Sugandhi N, Penazzato M. Performance of virological testing for early infant diagnosis: A systematic review. JAIDS J Acquir Immune Defic Syndr. 2017;160(2012):1.

17. Ciaranello AL, Lu Z, Ayaya S, Losina E, Musick B, Vreeman R, et al. Incidence of WHO Stage 3 and 4 Events, Tuberculosis, and Mortality in Untreated, HIV-Infected Children Enrolling in Care Before 1 Year of Age: An Iedea (International Epidemiologic Databases To Evaluate AIDS) East Africa Regional Analysis. Pediatr Infect Dis J. 2014;33(6):623–9.

18. Holmes C, Wood R, Badri M. CD4 decline and incidence of opportunistic infections in Cape Town, South Africa: Implications for prophylaxis and treatment. J Acquir Immune Defic Syndr. 2006;42:464–9.

19. Francke JA, Penazzato M, Hou T, Abrams EJ, Maclean RL, Myer L, et al. Clinical Impact and Cost-effectiveness of Diagnosing HIV Infection during Early Infancy in South Africa: Test Timing and Frequency. J Infect Dis. 2016;214(9):1319–28.

20. Briggs A, Weinstein M, Fenwick E. Model Parameter Estimation and Uncertainty: A Report of the ISPOR-SMDM Modeling Good Research Practices Task Force-6. Value Heal. 2012;15:835–42.

21. Husereau D, Drummond M, Petrou S, Carswell C, Moher D, Greenberg D. Consolidated Health Economic Evaluation Reporting Standards (CHEERS)--Explanation and elaboration: A report of the ISPOR Health Economic Evaluations Publication Guidelines Task Force. Value Heal. 2013;16:231–50.

22. Iliff PJ, Piwoz EG, Tavengwa N V, Zunguza CD, Marinda ET, Nathoo KJ, et al. Early exclusive breastfeeding reduces the risk of postnatal HIV-1 transmission and increases HIV-free survival. Aids [Internet]. 2005;19(7):699–708. Available from: http://content.wkhealth.com/linkback/openurl?sid=WKPTLP:landingpage&an=00002030-200504290-00007

23. Lilian RR, Johnson LF, Moolla H, Sherman GG. A mathematical model evaluating the timing of early diagnostic testing in HIV-exposed infants in South Africa. J Acquir Immune Defic Syndr [Internet]. 2014;67(3):341–8. Available from: http://www.ncbi.nlm.nih.gov/pubmed/25118910

24. Kuhn L, Kroon M. Breastfeeding and the 2015 South African guidelines for prevention of mother-to-child transmission of HIV. S Afr J HIV Med. 2015;16(1), Art. #377, 5 pages. http://dx.doi.org/10.4102/sajhivmed.v16i1.377.

25. Shapiro RL, Hughes MD, Ogwu A, Kitch D, Lockman S, Moffat C, et al. Antiretroviral Regimens in Pregnancy and Breast-Feeding in Botswana. N Engl J Med. 2010;362(24):2282–94.

26. Kesho Bora Study Group, de Vincenzi I. Triple antiretroviral compared with zidovudine and single-dose nevirapine prophylaxis during pregnancy and breastfeeding for prevention of mother-to-child transmission of HIV-1 (Kesho Bora study): A randomised controlled trial. Lancet Infect Dis [Internet]. 2011;11(3):171–80.

27. Fawzi W, Msamanga G, Spiegelman D, Renjifo B, Bang H, Kapiga S. Transmission of HIV-1 through breastfeeding among women in Dar es Salaam. J Acquir Immune Defic Syndr. 2002;31(3):331–8.

28. Dabis F, Bequet L, Ekouevi DK, Viho I, Rouet F, Horo A, et al. Field efficacy of zidovudine, lamivudine and single-dose nevirapine to prevent peripartum HIV transmission. AIDS. 2005;19(3):309–18.

29. Thior I, Lockman S, Smeaton LM, Shapiro RL, Wester C, Heymann SJ, et al. Breastfeeding Plus Infant Zidovudine Prophylaxis for 6 Months vs Formula Feeding Plus Infant Zidovudine for 1 Month to Reduce Mother-to-Child HIV Transmission in Botswana. JAMA. 2006;296(7):794.

30. Peltier CA, Ndayisaba GF, Lepage P, van Griensven J, Leroy V, Pharm CO, et al. Breastfeeding with maternal antiretroviral therapy or formula feeding to prevent HIV postnatal mother-to-child transmission in Rwanda. AIDS. 2009;23(April):2415–23.

31. Palombi L, Marazzi MC, Voetberg A, Magid NA. Treatment acceleration program and the experience of the DREAM program in prevention of mother-to-child transmission of HIV. AIDS. 2007;21(Suppl 4):S65–71.

32. Chasela C, Hudgens M, Jamieson D, Kayira D, Hosseinipour M, Kourtis A. Maternal or infant antiretroviral drugs to reduce HIV-1 transmission. N Engl J Med. 2010;362(24):2271–81.

33. Vyankandondera J, Luchters S, Hassink E. Reducing risk of HIV-1 transmission from mother to infant through breastfeeding using antiretroviral prophylaxis in infants. In: International AIDS Society Conference on HIV pathogenesis, treatment and prevention. p. (SIMBA-study, Abstract N°LB7).

34. Thomas T, Masaba R, Borkowf C, Ndivo R, Zeh C, Misore A. Triple-antiretroviral prophylaxis to prevent mother-to-child HIV transmission through breastfeeding--the Kisumu Breastfeeding Study, Kenya: a clinical trial. PLoS Med. 2011;8(3):e1001015.

35. Kuhn L, Aldrovandi GM, Sinkala M, Kankasa C, Semrau K, Mwiya M, et al. Effects of Early, Abrupt Weaning on HIV-free Survival of Children in Zambia. N Engl J Med. 2008;359(2):130–41.

36. World Health Organization. Progress report on the global plan towards the elimination of new HIV infections among children and keeping their mothers alive [Internet]. Geneva, Switzerland; 2015. Available from: http://www.unaids.org/sites/default/files/media_asset/JC2774_2015ProgressReport_GlobalPlan_en.pdf.

37. Ciaranello A, Myer L, Kelly K, Christensen S, Daskilewicz K, Doherty K. The cost-effectiveness of point of care CD4 testing in antenatal care in South Africa. PLoS One. 2015;March(5):In Press.

38. Marston M, Becquet R, Zaba B, Moulton LH, Gray G, Coovadia H, et al. Net survival of perinatally and postnatally HIV-infected children: A pooled analysis of individual data from sub-Saharan Africa. Int J Epidemiol. 2011;40(2):385–96.

39. Becquet R, Marston M, Dabis F, Moulton LH, Gray G, Coovadia HM, et al. Children who acquire hiv infection perinatally are at higher risk of early death than those acquiring infection through breastmilk: A meta-analysis. PLoS One. 2012;7(2).

40. United Nations. World Population Prospects: The 2008 Revision. New York, USA;

41. Mallampati D, Ford N, Hanaford A, Sugandhi N, Penazzato M. Performance of virological testing for early infant diagnosis: A systematic review. J Acquir Immune Defic Syndr. 2017;160(2012):1.

42. Babiker A, Castro nee Green H, Compagnucci A, Fiscus S, Giaquinto C, Gibb D. First-line antiretroviral therapy with a protease inhibitor versus non-nucleoside reverse transcriptase inhibitor and switch at higher versus low viral load in HIV-infected children: an open-label, randomised phase 2/3 trial. Lancet Infect Dis. 2011;11(4):273–83.

43. Tuboi S, Brinkhof M, Egger M, Stone R, Braistein P, Nash D. Discordant responses to potent antiretroviral treatment in previously naive HIV-1 infected adults initiating treatment in resource-constrained countries: the antiretroviral threapy in low income counties collaboration. J Acquir Immune Defic Syndr. 2007;45(1):52–9.

44. Ciaranello AL, Chang Y, Margulis A V, Bassett I V, Losina E, Rochelle P. Effectiveness of Pediatric Antiretroviral Therapy in Resource-limited Settings: A Systematic Review and Meta-analysis. Clin Infect Dis. 2009;49(12):1915–27.

45. Sutcliffe CG, van Dijk JH, Bolton C, Persaud D, Moss WJ. Effectiveness of antiretroviral therapy among HIV-infected children in sub-Saharan Africa. Lancet Infect Dis. 2008;8(8):477–89.

46. Thomas L. Costing of HIV/AIDS services at a tertiary level hospital in Gauteng Province. University of Witwatersrand, South Africa; 2006.

47. Goldie SJ, Yazdanpanah Y, Losina E, Weinstein MC, Anglaret X, Walensky RP, et al. Cost-Effectiveness of HIV Treatment in Resource-Poor Settings — The Case of Côte d’Ivoire. N Engl J Med [Internet]. 2006;355(11):1141–53. Available from: http://www.nejm.org/doi/abs/10.1056/NEJMsa060247

48. Cleary S, Okorafor OA, Chitha W, Boulle A, Jikwana S. Financing antiretroviral treatment and primary health care services. South African Heal Rev. 2005;58–74.

49. Clinton Health Access Initiative. 2016 Antiretroviral (ARV) CHAI reference price list. 2016;(November):2015–6. Available from: http://www.clintonhealthaccess.org/content/uploads/2016/11/2016-CHAI-ARV-Reference-Price-List_FINAL.pdf (Accessed March 03, 2017)

50. Doherty K, Essajee S, Penazzato M, Holmes C, Resch S, Ciaranello A. Estimating age-based antiretroviral therapy costs for HIV-infected children in resource-limited settings based on World Health Organization weight-based dosing recommendations. BMC Health Serv Res [Internet]. 2014;14(1):201. Available from: http://bmchealthservres.biomedcentral.com/articles/10.1186/1472-6963-14-201

51. Bassett I V, Giddy J, Nkera J, Wang B, Losina E, Lu Z, et al. Routine voluntary HIV testing in Durban, South Africa: the experience from an outpatient department. J Acquir Immune Defic Syndr. 2007;46(2):181–6.
